# Hypervalent Iodine-Mediated Synthesis of Steroidal 5/5-Spiroiminals

**DOI:** 10.3390/molecules29235812

**Published:** 2024-12-09

**Authors:** Rayala Naveen Kumar, Seongmin Lee

**Affiliations:** The Division of Chemical Biology and Medicinal Chemistry, College of Pharmacy, University of Texas at Austin, Austin, TX 78712, USA; navirayala@gmail.com

**Keywords:** hypervalent iodine, spiroiminal, C-H activation, Steroids

## Abstract

The hypervalent iodine-mediated formation of steroidal 5/5-spiroiminals and 5/5-spiroaminals from steroidal amines is presented. Under the influence of excess PhI(OAc)_2_ and iodine in acetonitrile at 0 °C, steroidal amines smoothly underwent cyclization to give a mixture of 5/5-spiroiminals and 5/5-spiroaminals. This reaction represents the first example of a C-H-activation-mediated formation of a spiroiminal. Presumably, the formation of 5/5-spiroiminals occurs through aminyl radical-mediated cyclization followed by amine-to-imine oxidation.

## 1. Introduction

**Figure 1:**
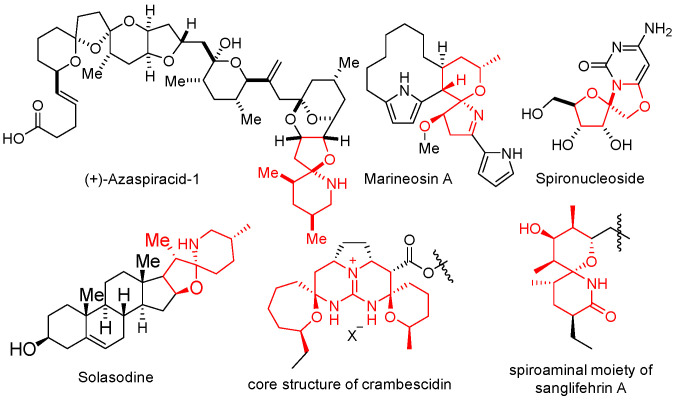
Representative natural products containing spiroaminal and spiroiminal moieties. The spiroaminal and spiroiminal moieties of the natural products are shown in red.

Spiroaminal and spiroiminal moieties are found in a wide variety of natural products with biological activities. For example, steroidal spiroaminals, such as solasodine, solamargine, and tomatidenol, have shown potent antitumor and anti-inflammatory activities (Figure 1) [1,2,3,4,5,6,7]. Solamargine, which possesses a 5/6-spiroaminal moiety, is a glycoside solasodine that has shown high efficacy in treating advanced melanomas [8,9,10]. Marineosin A containing a 5/6-spiroiminal moiety shows broad antitumor activity [11,12,13]. Sanglifehrin A bearing a 6/6-spiroaminal moiety exhibits immunosuppressive activity [14,15,16,17]. Crambescidin alkaloids containing a 6/6-spiroaminal moiety display nanomolar cytotoxicity against several tumor cell lines [18,19,20].

For the synthesis of spiroaminal/spiroiminal-containing natural products and their analogs, efficient synthetic methods for the construction of spiroiminal/spiroaminal moieties would be useful. The establishment of a spiroaminal moiety has been achieved through various methods, including the cyclocondensation of ketone alkanolamines [21] and Au(I)-catalyzed cycloisomerization of alkyne alkanolamines [22,23,24]. The majority of existing methods for the synthesis of 5/6-spiroaminals involves the use of acid-catalyzed cyclization reactions [25,26]. In the literature, some of the approaches to the synthesis of spiroaminals through the ring-closing of *N*-exocyclic hemiaminals have been described [27]. Another approach to the 1,3-dipolar cyclization of azomethine ylides for the construction of [5,5]-spiroaminals was accomplished by Fishwick et al. [28,29,30]. Monocondensed aromatic spiropyrans were attained by the addition of Fisher base and substituted salicylaldehyde [31,32]. Bermejo et al. were able to synthesize spiroaminals through an NIS-based oxidative cyclization of *N*-substituted tetrahydropyridines [33,34]. Kende et al. described the synthesis of spiroaminal compounds by *N*-acyliminium cyclization chemistry [35]. Huang et al. achieved aza-spiropyrans through the addition of organometallic bromide to succinimides [36]. In azaspiracid synthesis, Carter et al. achieved an aza-spiro precursor using Yb(OTf)_3_ in a stereoselective manner [37]. Suarez et al. were the first to envisage hypervalent iodide-mediated radical cyclization for the synthesis of oxa–aza spirocyclic compounds where *N*-phosphoramidate and *N*-cyanamide were used [38,39,40]. Forsyth et al. developed the one-pot Staudinger reduction/intramolecular aza-Wittig imine capture sequence for the synthesis of spiroaminals [41]. Xu et al. introduced the spiroiminal moiety of marineosin A via spirolactam formation followed by a Vilsmeier–Haack-type reaction [12].

**Scheme 1:**
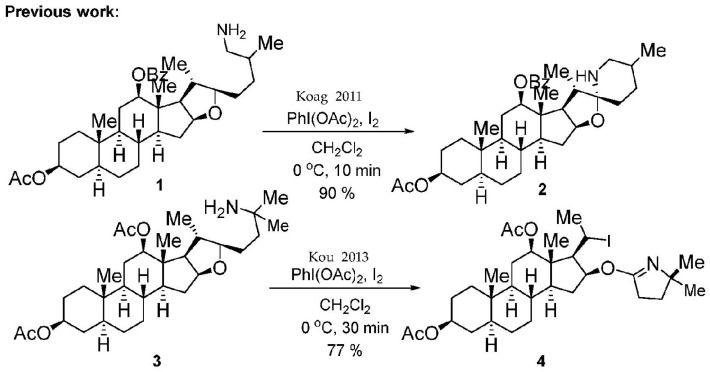
Our previous studies of PhI(OAc)_2_/I_2_-mediated cyclization of steroidal amines [42,43].

We have previously reported the hypervalent iodine-mediated cyclization of a steroidal primary amine (**1**) where a steroidal 5/6-spiroaminal (**2**) formed under the influence of PhI(OAc)_2_ and I_2_, presumably via an aminyl radical cyclization pathway [42]. When a steroidal amine (**3**) was subjected to the same reaction conditions, an E-ring-opened oxyimine (**4**) formed exclusively, presumably via aminyl radical cyclization followed by an oxidative E-ring opening (Scheme 1) [43]. Observation of the formation of a 5/6 spiroaminal (**2**) and E-ring-opened oxyimine (**4**) from steroidal primary amines, respectively, has prompted us to further explore the substrate dependency of the hypervalent iodine-mediated cyclization of alkylamines. Herein, we report the formation of 5/5-spiroiminals and spiroaminals from steroidal alkylamines in the presence of PhI(OAc)_2_ and I_2_.

## 2. Results and Discussion

**Scheme 2:**
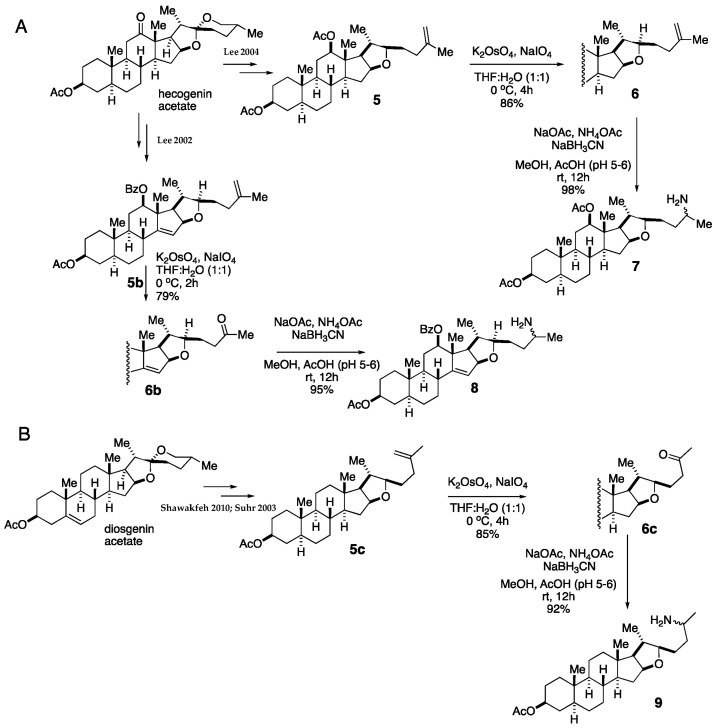
Preparation of steroidal amines (**7**), (**8**), and (**9**) from steroidal spiroketals. (**A**) Syntheses of steroidal amines (**7**) and (**8**) from hecogenin acetate. (**B**) Synthesis of steroidal amine (**9**) from diosgenin acetate [44,45,46,47].

Preparation of the steroidal amine (**7**) started with the synthesis of a terminal alkene (**5)** from a hecogenin acetate by using previously reported procedures (Scheme 2). The oxidative opening of a 2,2-disubstituted alkene (**5**) with K_2_OsO_4_/NaIO_4_ yielded a C25-ketone (**6)** with an 86% yield. The ketone (**6**) was converted into a C25-amine (**7**) via reductive amination (Scheme 2A). Steroidal amines (**8**) and (**9**) were prepared from the corresponding ketones (**6b**) and (**6c**), respectively, via reductive amination.

**Scheme 3:**
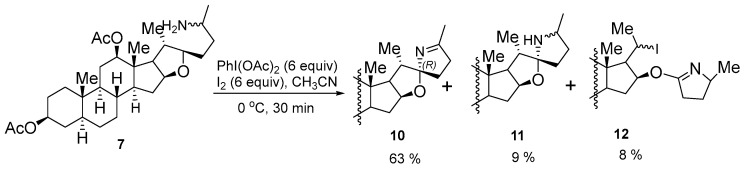
PhI(OAc)_2_/I_2_-mediated cyclization of steroidal amine (**7**).

**Figure 2:**
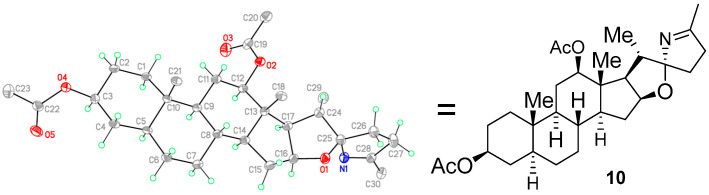
ORTEP representation of 5/5-spiroiminal steroid (**10**).

With the steroidal amines (**7**–**9**) in hand, we next evaluated the substrate dependency of hypervalent iodine-mediated cyclization. Under the influence of PhI(OAc)_2_ and I_2_ in acetonitrile at 0 °C, the hecogenin-derived amine (**7**) underwent facile oxidation, yielding a mixture of three products (Scheme 3) rather than the single product previously observed with steroidal amines (Scheme 1). Interestingly, in addition to the 5/5-spiroaminal (**11**) and the E-ring-opened oxyimine (**12**), a 5/5-spiroiminal compound (**10**) formed as the major product. The steroidal 5/5-spiroiminal (**10**) contained a C22-*R* stereochemistry and a double bond between the C25 and the nitrogen atom. The chemical structure and stereochemistry of the 5/5-spiroiminal (**10**) was unambiguously determined by single-crystal X-ray crystallography (Figure 2, CCDC No. 1573262). The exclusive formation of a C22-*R* spiroiminal, as opposed to a C22-*S* spiroiminal, indicates that the reaction is thermodynamically controlled.

The use of an acetonitrile solvent and excess PhI(OAc)_2_/I_2_ promoted the formation of a spiroiminal compound as the major product (Table 1, entries 1–3). The use of other solvents, such as CH_2_Cl_2_, tetrahydrofuran, and benzene, resulted in a decreased yield of the spiroiminal (**10**). When tetrahydrofuran was used for the reaction, an E-ring-opened oxyimine (**12**) was produced as the major product, highlighting the effect of the solvent on product distribution. Notably, the cyclization of the steroidal amine (**7**) did not occur in dioxane. When optimized conditions (6 molar equiv. of PhI(OAc)_2_/I_2_, MeCN, 0 °C, 30 min.) were used for the cyclization of steroidal primary amines (**1**) and (**3**), the 5/6-spiroaminal (**2**) and E-ring-opened oxyimine (**4**), respectively, were exclusively formed. This highlights that the hypervalent iodine-mediated cyclization of the steroidal amines is substrate-dependent. The substitution at the Cα position of the amine (e.g., CH_2_, C(CH_3_)_2_) greatly affects the PhI(OAc)_2_/I_2_-mediated cyclization of steroidal amines, leading to varying product distribution of spiroaminals, spiroiminals, and oxyimines.

Previous spiroiminal syntheses have involved a conversion from spirolactam, methoxy imine, or hemiacetal. [12] To the best of our knowledge, this reaction represents the first example of the C-H-activation-mediated formation of a spiroiminal.

With optimized cyclization conditions, we surveyed hypoiodite-mediated oxidation in other steroidal amines (Table 2). Treatment of the Δ^14^ olefin-containing steroidal amine (**8**) and diosgenin-derived amine (**9**) with PhI(OAc)_2_/I_2_ gave rise to a mixture of 5/5-spiroiminal, 5/5-spiroaminal, and iodocyclic ethers. It is noteworthy that the C14–15 double bond does not undergo oxidation under these conditions (Entry 1, Table 2).

**Scheme 4:**
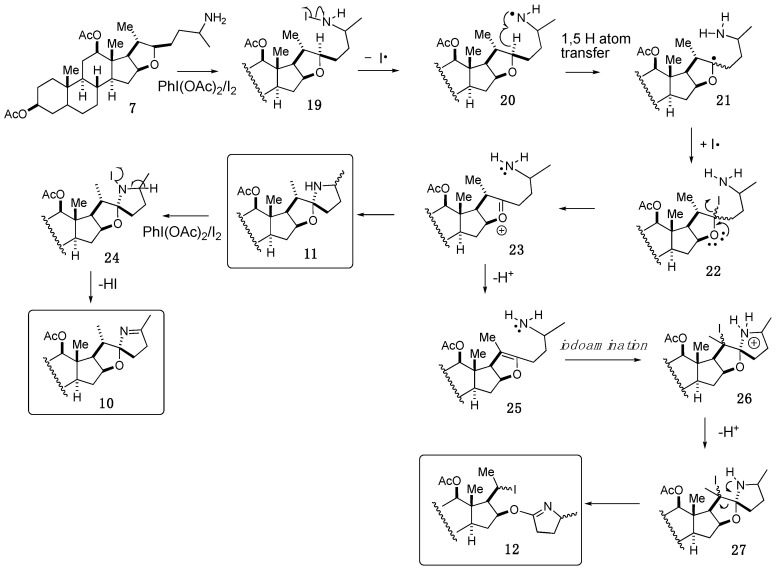
A plausible mechanism for PhI(OAc)_2_/I_2_-mediated formation of a spiroiminal (**10**), spiroaminal (**11**), and oxyimine (**12**).

A plausible mechanism for the formation of a spiroiminal (**10**), spiroaminal (**11**), and oxyimine (**12**) is outlined in Scheme 4. The reaction of PhI(OAc)_2_ and I_2_ with the steroidal amine (**7**) generates an *N*-iodoamine (**19**). Homolytic cleavage of the N–I bond in (**19**) produces an aminyl radical (**20**), which undergoes a 1,5-hydrogen atom transfer to form a carbon radical (**21**). This radical is stabilized by the adjacent oxygen atom in the tetrahydrofuran ring. The subsequent reaction of the carbon radical with an iodine radical yields an iodoether (**22**), which undergoes oxygen-assisted iodine elimination to form an oxacarbenium ion (**23**). An intramolecular nucleophilic attack by the primary amine on an oxacarbenium ion (**23**) generates a spiroaminal (**11**). Further oxidation of the spiroaminal (**11**) by PhI(OAc)_2_/I_2_ produces a chemically unstable *N*-iodoamine (**24**), which undergoes elimination to form a spiroiminal (**10**). Alternatively, the oxacarbenium ion (**23**) can be transformed into a cyclic enol ether (**25**). A ring-closing iodoamination of this intermediate forms a sterically congested iodospiroaminal (**26**), which undergoes fragmentation to produce an E-ring-opened oxyimine (**12**).

## 3. Materials and Methods

### 3.1. General Methods

Iodine and iodobenzene diacetate were purchased from Acros Organics. Solvents (dichloromethane, tetrahydrofuran, methanol, and dimethylformamide) were purchased from Fisher Chemical (Fair Lawn, NJ, USA). Triphenylphosphine and sodium azide were purchased from Alfa Aesar (Ward Hill, MA, USA). All reactions were conducted under a positive pressure of argon in anhydrous solvents. Reaction progress was monitored using thin-layer chromatography (TLC) on Silica Gel 60 F254 glass plates (EMD Chemicals Inc., Darmstadt, Germany) with the appropriate solvent systems for development. The TLC plates were visualized under ultraviolet light (254 nm) and with p-anisaldehyde staining. Analytical samples were purified using flash silica gel chromatography. ^1^H and ^13^C NMR spectra were recorded on a Varian MERCURY 400 (400 MHz) (International Equipment Trading Ltd., Mundelein, IL, USA). High-resolution mass spectrometry data were obtained using an Agilent 6530 Accurate-Mass Q-TOF LC/MS (Agilent, Santa Clara, CA, USA). 

### 3.2. Chemical Synthesis

#### 3.2.1. General Procedure for the Synthesis of Steroidal Ketone

A steroidal terminal alkene (**5**) was prepared in 5 steps from a hecogenin acetate [44,45,46,47]. Potassium osmate (VI) dihydrate (64 mg, 0.2 mmol) and NaIO_4_ (8.5 g, 40 mmol) were added to a solution of 1,2-disubstituted alkene (**5**) (5 g, 10 mmol) in tetrahydrofuran (100 mL) and water (100 mL) at 0 °C and the mixture was stirred at 0 °C for 4 h. After quenching the reaction with sodium sulfite, the reaction mixture was diluted with water (100 mL) and extracted with ethyl acetate (3 × 150 mL). The organic layer was washed with saturated aqueous sodium chloride, dried over anhydrous sodium sulfate, evaporated under reduced pressure, and purified by a silica gel column chromatography (petroleum ether-EtOAc, 7:3) to obtain the C25-ketone (**6**) (4.32 g, 86%) as a white solid. *R_f_* = 0.30 (petroleum ether-EtOAc, 4:1). Other ketone substrates (**6b**) and (**6c**) were also prepared by the same procedure (Scheme 2 and Supplementary Materials).

#### 3.2.2. 3β,12β-Diacetoxy-25-keto-5α-furostane (**6**)

^1^H NMR (400 MHz, CDCl_3_) δ 4.66 (tt, *J* = 10.9, 4.9 Hz, 1H), 4.55 (dd, *J* = 11.3, 4.7 Hz, 1H), 4.26 (td, *J* = 7.2, 4.9 Hz, 1H), 3.27 (td, *J* = 8.5, 2.7 Hz, 1H), 2.65 (dd, *J* = 19.4, 4.9 Hz, 1H), 2.49 (dd, *J* = 17.6, 6.0 Hz, 1H), 2.13 (s, 3H), 2.02 (s, 3H), 2.00 (s, 6H), 1.96–1.86 (m, 1H), 1.82–1.57 (m, 9H), 1.54–1.41 (m, 2H), 1.40–1.21 (m, 6H), 1.20–1.09 (m, 2H), 1.08–0.95 (m, 2H), 0.92 (d, *J* = 10.9, 3H), 0.85 (s, 3H), 0.83 (s, 3H). ^13^C NMR (100 MHz, CDCl_3_) δ 208.76, 170.63, 170.51, 89.01, 82.96, 81.40, 73.39, 63.89, 55.17, 52.68, 44.83, 44.47, 40.86, 38.57, 36.57, 35.57, 34.23, 33.80, 31.54, 29.95, 28.30, 27.28, 27.06, 26.67, 21.44, 21.42, 17.75, 12.10, 11.70. MS (ESI): *m*/*z* = 503 [M + H]^+^, 525 [M + Na]^+^. HRMS: calcd. for C_30_H_46_O_6_Na [M + Na]^+^: 525.3187: found: 525.3188.

#### 3.2.3. 3β-Acetoxy-12β-benzoyloxy-25-keto-5α-furostan-14-ene (**6b**)

^1^H NMR (400 MHz, CDCl_3_) δ 8.06–7.99 (m, 2H), 7.59–7.52 (m, 1H), 7.48–7.40 (m, 2H), 5.45 (t, *J* = 2.0 Hz, 1H), 4.75 (dd, *J* = 8.1, 2.3 Hz, 1H), 4.67 (dt, *J* = 11.2, 4.0 Hz, 2H), 4.27 (td, *J* = 6.4, 0.9 Hz, 1H), 3.21 (td, *J* = 9.3, 2.5 Hz, 1H), 2.69 (dd, *J* = 9.8, 5.1 Hz, 1H), 2.49 (dd, *J* = 17.9, 6.0 Hz, 1H), 2.23 (t, *J* = 7.9 Hz, 1H), 2.11 (s, 3H), 1.99 (s, 3H), 2.05–1.87 (m, 3H), 1.83–1.76 (m, 1H), 1.76–1.44 (m, 7H), 1.42–1.12 (m, 5H), 1.21 (s, 3H), 1.11–0.97 (m, 1H), 0.87 (s, 3H), 0.83 (d, *J* = 10.8, 3H). ^13^C NMR (101 MHz, CDCl_3_) δ 208.71, 170.63, 165.98, 160.97, 157.38, 133.02, 130.38, 129.44, 128.43, 119.90, 86.09, 85.98, 81.67, 73.06, 59.48, 51.95, 44.18, 41.11, 40.71, 36.53, 35.91, 33.73, 29.98, 29.49, 28.03, 27.23, 27.00, 26.88, 26.65, 21.40, 16.60, 15.97, 11.97. MS (ESI): *m*/*z* = 585 [M + Na]^+^. HRMS: calcd. for C_35_H_46_O_6_Na [M + Na]^+^: 585.3187: found: 585.3191.

#### 3.2.4. 3β-Acetoxy-25-keto-5α-furostane (**6c**)

^1^H NMR (400 MHz, CDCl_3_) δ 4.67 (tt, *J* = 11.3, 4.9 Hz, 1H), 4.26 (td, *J* = 7.8, 5.2 Hz, 1H), 3.28 (td, *J* = 8.8, 3.0 Hz, 1H), 2.66 (dd, *J* = 9.7, 5.3 Hz, 1H), 2.49 (dd, *J* = 17.7, 6.0 Hz, 1H), 2.13 (s, 3H), 2.05–1.86 (m, 4H), 1.83–1.41 (m, 12H), 1.40–0.93 (m, 9H), 0.97 (s, 3H), 0.92–0.56 (m, 2H), 0.82 (s, 3H), 0.75 (s, 3H). ^13^C NMR (100 MHz, CDCl_3_) δ 208.85, 170.69, 89.05, 83.29, 73.64, 65.07, 56.64, 54.20, 44.62, 40.96, 40.90, 39.57, 37.82, 36.72, 35.54, 35.21, 33.97, 32.07, 29.95, 28.44, 27.43, 27.02, 21.46, 20.78, 18.61, 16.58, 12.24. MS (ESI): *m*/*z* = 467.3 [M + Na]^+^.

#### 3.2.5. General Procedure for the Synthesis of Steroidal Amines (**7**)

Ammonium acetate (24.6 g, 320 mmol) and sodium acetate (656 mg, 8 mmol) were added to a solution of steroidal C25-ketone (**6**) (4.02 g, 8 mmol) in 100 mL MeOH. After stirring for 10 min at 25 °C, sodium cyanoborohydride (3.01 g, 48 mmol) was added portion-wise to the reaction mixture at 25 °C. The pH of the reaction was adjusted to 5–6 by adding glacial acetic acid. After stirring overnight at the same temperature, a MeOH solvent of the reaction mixture was removed under reduced pressure. Water (300 mL) was added to the residue and the pH of the reaction mixture was adjusted to 9–10 by slowly adding 1 N NaOH. The reaction mixture was then diluted with EtOAc (200 mL) and the aqueous layer was extracted with EtOAc (3 × 150 mL). The combined organic layers were dried over anhydrous Na_2_SO_4_ and concentrated under reduced pressure to obtain the steroidal amine (**7**) (3.9 g, 98%) as a white solid. *R_f_* = 0.30 (MeOH-EtOAc = 1:4). Steroidal amines (**8**) and (**9**) were also prepared following the procedure described above.

#### 3.2.6. 3β,12β-Diacetoxy-25-amino-5α-furostane (**7**)

^1^H NMR (400 MHz, CHCl_3_) δ 4.66 (tt, *J* = 10.9, 4.9 Hz, 1H, C3-H), 4.55 (dd, *J* = 11.2, 4.6 Hz, 1H, C12-H), 4.28 (td, *J* = 7.7, 5.2 Hz, 1H, C16-H), 3.30 (dd, *J* = 11.3, 6.5 Hz, 1H, C22-H), 2.93–2.84 (m, 1H), 2.01 (d, *J* = 8.8 Hz, 9H), 1.83–1.44 (m, 10H), 1.43–1.22 (m, 6H), 1.15 (dd, *J* = 7.8, 5.4 Hz, 4H), 1.10–1.01 (m, 4H), 0.94–0.79 (m, 11H). ^13^C NMR (100 MHz, CDCl_3_) δ 170.64, 170.46, 89.62, 83.51, 81.29, 73.35, 63.32, 60.39, 55.14, 52.61, 49.32, 44.81, 44.44, 38.79, 36.53, 35.55, 34.15, 33.76, 32.05, 28.26, 27.25, 26.64, 21.40, 19.80, 18.32, 17.66, 14.16, 12.09, 11.80. MS (ESI): *m*/*z* = 504 [M + H]^+^. HRMS: calcd. for C_30_H_49_NO_5_ [M + H]^+^: 504.3688: found: 504.3684.

#### 3.2.7. 3β-Acetoxy-12β-benzoyloxy-25-amino-5α-furostan-14-ene (**8**)

^1^H NMR (400 MHz, CDCl_3_) δ 8.09–7.98 (m, 2H), 7.61–7.52 (m, 1H), 7.50–7.39 (m, 2H), 5.47 (t, *J* = 2.0 Hz, 1H), 4.86–4.72 (m, 1H), 4.69 (dt, *J* = 10.6, 5.2 Hz, 2H), 3.31–3.11 (m, 1H), 2.86 (m, 1H), 2.22 (q, *J* = 7.2, 6.5 Hz, 1H), 2.11 (q, *J* = 10.2, 7.0 Hz, 1H), 2.06–1.85 (m, 6H), 1.84–1.45 (m, 10H), 1.38–1.13 (m, 9H), 1.11–0.79 (m, 11H). ^13^C NMR (100 MHz, CDCl_3_) δ 170.60, 165.95, 157.24, 157.21, 132.99, 130.44, 129.45, 128.42, 120.09, 87.00, 86.01, 81.75, 73.25, 59.61, 51.94, 47.18, 44.18, 41.17, 37.15, 37.01, 36.54, 35.91, 34.16, 33.74, 30.19, 29.49, 28.04, 27.24, 26.67, 23.95, 21.40, 16.83, 16.01, 11.98. MS (ESI): *m*/*z* = 564 [M + H]^+^. HRMS: calcd. for C_35_H_50_NO_5_ [M + H]^+^: 564.3684: found: 564.3696.

#### 3.2.8. 3β-Acetoxy-25-amino-5α-furostane (**9**)

^1^H NMR (400 MHz, CDCl_3_) δ 4.66 (tt, *J* = 11.3, 4.9 Hz, 1H), 4.28 (m, 1H), 3.38–3.24 (m, 1H), 3.00–2.86 (m, 1H), 2.68 (s, 2H), 2.00 (s, 4H), 1.83–1.39 (m, 12H), 1.38–1.20 (m, 6H), 1.17–0.93 (m, 10H), 0.92–0.56 (m, 9H). ^13^C NMR (100 MHz, CDCl_3_) δ 170.69, 90.07, 83.31, 73.64, 65.09, 56.64, 54.20, 47.53, 47.03, 44.62, 40.96, 39.58, 37.98, 36.71, 35.53, 33.96, 32.07, 30.36, 29.92, 28.44, 27.43, 23.24, 21.45, 20.78, 18.85, 16.60, 12.24. MS (ESI): *m*/*z* = 446.3 [M + H]^+^.

#### 3.2.9. Synthesis of 5/5-Spiroiminals and 5/5-Spiroaminals (**10**, **13,** and **16**)

The C25-amine (**7**) (445 mg, 1 mmol) at 0 °C was added to a CH_3_CN (10 mL) solution of PhI(OAc)_2_ (1.93 g, 6 mmol) and iodine (1.52 g, 6 mmol) and the resulting mixture stirred vigorously at the same temperature. After a complete conversion of the starting material, the reaction mixture was quenched by adding saturated aqueous Na_2_S_2_O_3_ solution and extracted with CH_2_Cl_2_ (3 × 50 mL). The organic layer was washed with brine, dried over anhydrous sodium sulfate, concentrated, and subjected to silica gel column chromatography (MeOH:EtOAc = 1:19) to obtain a 5/5-spiroiminal (**10**) (314 mg, 63%), 5/5-spiroaminal (**11**) (45 mg, 9%), and E-ring-opened oxyimine (**12**) (50 mg, 8%) as a white solid. Spiroiminals (**13**) and (**16**) were also prepared from steroidal amines (**8**) and (**9**), respectively, following the same procedure (see Table 2).

#### 3.2.10. 3β,12β-Diacetoxy-25-imino-5α-spirostane (**10**)

^1^H NMR (400 MHz, CDCl_3_) δ 4.74–4.60 (m, 2H), 4.55 (dd, *J* = 11.2, 4.7 Hz, 1H), 2.59 (dd, *J* = 9.5, 6.0 Hz, 1H), 2.39 (dd, *J* = 9.6, 4.9 Hz, 1H), 2.19–1.93 (m, 13H), 1.82 (dt, *J* = 12.6, 4.6 Hz, 2H), 1.74–1.58 (m, 5H), 1.57–1.43 (m, 2H), 1.42–0.99 (m, 9H), 0.92 (s, 3H), 0.84 (s, 3H), 0.78 (d, *J* = 10.4, 3H). ^13^C NMR (100 MHz, CDCl_3_) δ 176.80, 170.60, 170.32, 118.47, 82.33, 81.69, 73.39, 61.52, 54.67, 52.55, 44.77, 44.45, 42.10, 38.17, 36.49, 35.59, 34.03, 33.79, 31.90, 31.59, 31.39, 28.31, 27.26, 26.69, 21.51, 21.41, 19.90, 13.37, 12.09, 11.82. MS (ESI): *m*/*z* = 500 [M + H]^+^. HRMS: calcd. for C_30_H_49_NO_5_ [M + H]^+^: 500.3371: found: 500.3371.

#### 3.2.11. 3β-Acetoxy-12β-benzoyloxy-25-imino-5α-spirostane (**13**)

^1^H NMR (500 MHz, CDCl_3_) δ 8.06–8.00 (m, 2H), 7.54 (t, *J* = 7.4 Hz, 1H), 7.44 (dt, *J* = 15.2, 7.6 Hz, 2H), 5.47 (t, *J* = 1.8 Hz, 1H), 5.19 (dd, *J* = 8.4, 2.1 Hz, 1H), 4.69 (tt, *J* = 10.9, 4.9 Hz, 1H), 4.61 (dd, *J* = 11.3, 4.7 Hz, 1H), 2.70 (t, *J* = 8.9 Hz, 1H), 2.61 (dd, *J* = 9.7, 5.7 Hz, 1H), 2.38 (dd, *J* = 9.8, 5.0 Hz, 1H), 2.19–2.11 (m, 2H), 2.07 (dt, *J* = 11.9, 8.6, Hz, 2H), 2.02 (d, *J* = 3.9 Hz, 4H), 2.00–1.77 (m, 4H), 1.75–1.43 (m, 5H), 1.42–1.15 (m, 4H), 1.13–0.99 (m, 3H), 0.89 (s, 6H), 0.82 (d, *J* = 6.9 Hz, 3H). ^13^C NMR (100 MHz, CDCl_3_) δ 176.89, 170.60, 165.74, 156.04, 132.77, 130.68, 129.42, 128.29, 120.69, 115.97, 86.14, 81.50, 73.28, 56.16, 52.18, 51.65, 44.39, 38.29, 36.45, 34.64, 33.76, 31.57, 29.47, 28.09, 27.24, 26.42, 25.26, 22.64, 21.41, 19.88, 14.72, 13.35, 11.98. MS (ESI): *m*/*z* = 560 [M + H]^+^. HRMS: calcd. for C_35_H_46_NO_5_ [M + H]^+^: 560.3376: found: 560.3395.

#### 3.2.12. 3β-Acetoxy-25-imino-5α-spirostane (**16**)

^1^H NMR (400 MHz, CDCl_3_) δ 4.77–4.57 (m, 2H), 2.59 (dd, *J* = 9.5, 6.0 Hz, 1H), 2.38 (dd, *J* = 9.6, 4.9 Hz, 1H), 2.21–1.90 (m, 9H), 1.85–1.43 (m, 6H), 1.40–1.23 (m, 9H), 1.24 (s, 3H), 1.04–0.85 (m, 2H), 0.84 (d, *J* = 12.4, 3H), 0.82 (s, 6H). ^13^C NMR (100 MHz, CDCl_3_) δ 176.52, 170.68, 118.49, 82.69, 73.66, 62.51, 56.19, 54.10, 44.59, 41.65, 40.79, 40.01, 38.17, 36.66, 35.56, 35.03, 33.98, 32.10, 31.99, 29.69, 28.45, 27.44, 21.46, 20.99, 19.94, 16.67, 14.17, 12.24. MS (ESI): *m*/*z* = 442 [M + H]^+^. HRMS: calcd. for C_28_H_44_NO_3_ [M + H]^+^: 442.3316: found: 442.3328.

#### 3.2.13. 3β,12β-Diacetoxy-25-amino-5α-spirostane (**11**)

^1^H NMR (400 MHz, CDCl_3_) δ 8.01 (s, 1H), 4.65 (tt, *J* = 10.9, 4.9 Hz, 1H), 4.58–4.49 (m, 1H), 4.36–4.24 (m, 1H), 2.53 (dt, *J* = 16.8, 9.1 Hz, 1H), 2.32–2.07 (m, 4H), 2.04–1.97 (m, 7H), 1.88–1.68 (m, 3H), 1.72–1.40 (m, 7H), 1.37–0.99 (m, 11H), 0.85 (dd, *J* = 19.1, 6.1 Hz, 10H). ^13^C NMR (100 MHz, CDCl_3_) δ 178.95, 170.61, 103.67, 81.38, 80.82, 77.34, 73.34, 60.78, 60.38, 54.65, 52.57, 44.99, 44.43, 40.23, 36.52, 35.56, 34.08, 33.76, 31.54, 31.20, 29.70, 28.24, 27.25, 26.69, 21.50, 21.41, 14.44, 14.18, 12.08. MS (ESI): *m*/*z* = 502 [M + H]^+^. HRMS: calcd. for C_30_H_47_NO_5_ [M + H]^+^: 502.3527: found: 502.3517.

#### 3.2.14. 3β-Acetoxy-12β-benzoyloxy-25-amino-5α-spirostane (**14**)

^1^H NMR (400 MHz, CDCl_3_) δ 8.07–7.96 (m, 2H), 7.61–7.51 (m, 1H), 7.50–7.40 (m, 2H), 6.42 (s, 1H), 5.43 (q, *J* = 2.1 Hz, 1H), 4.89–4.80 (m, 1H), 4.77–4.62 (m, 1H), 2.53 (dt, *J* = 16.4, 8.8 Hz, 1H), 2.32 (dd, *J* = 9.8, 1.7 Hz, 1H), 2.28–2.06 (m, 5H), 2.01 (s, 3H), 1.93–1.76 (m, 3H), 1.74–1.55 (m, 6H), 1.53–1.13 (m, 11H), 1.12–0.99 (m, 2H), 0.90–0.83 (m, 6H). ^13^C NMR (100 MHz, CDCl_3_) δ 177.83, 170.61, 165.98, 156.90, 133.15, 129.44, 128.49, 119.81, 100.83, 84.49, 81.02, 73.19, 56.02, 52.25, 52.05, 44.20, 42.21, 36.49, 35.97, 34.11, 33.71, 30.63, 29.46, 29.38, 27.97, 27.22, 26.66, 21.40, 15.30, 14.51, 11.98. MS (ESI): *m*/*z* = 584.3 [M + Na]^+^.

#### 3.2.15. 3β-Acetoxy-25-amino-5α-spirostane (**17**)

^1^H NMR (400 MHz, CDCl_3_) δ 5.79 (s, 1H), 4.68 (tt, *J* = 10.9, 4.7 Hz, 2H), 4.18–4.01 (m, 1H), 2.73–2.45 (m, 2H), 2.40–2.27 (m, 2H), 2.13–1.91 (m, 7H), 1.76 (dt, *J* = 13.4, 3.5 Hz, 4H), 1.69–1.45 (m, 9H), 1.43–1.08 (m, 11H), 1.05–0.73 (m, 6H). ^13^C NMR (100 MHz, CDCl_3_) δ 172.32, 171.41, 109.27, 81.54, 73.42, 72.63, 60.54, 55.11, 54.19, 44.69, 41.62, 41.02, 39.78, 38.18, 36.75, 35.49, 35.02, 33.94, 32.16, 30.61, 28.44, 26.47, 21.45, 20.92, 19.93, 17.62, 14.47, 11.23. MS (ESI): *m*/*z* = 444.3 [M + H]^+^.

#### 3.2.16. 3β,12β-Diacetoxy-16β-((2-methyl-3,4-dihydro-2H-pyrrol-5-yl)oxy)-17β-(1-iodoethyl)-5α-androstane (**12**)

^1^H NMR (400 MHz, CDCl_3_) δ 5.12 (dt, *J* = 7.7, 3.9 Hz, 1H), 4.66 (dd, *J* = 10.7, 4.7 Hz, 2H), 4.59–4.45 (m, 1H), 3.96 (m, 1H), 2.67–2.29 (m, 4H), 2.20–2.06 (m, 4H), 2.01 (td, *J* = 3.7, 3.3, 1.8 Hz, 4H), 1.83–1.74 (m, 2H), 1.71–1.40 (m, 9H), 1.40–1.08 (m, 9H), 1.03–0.72 (m, 9H). ^13^C NMR (100 MHz, CDCl_3_) δ 174.55, 171.21, 170.90, 170.60, 81.02, 80.92, 79.72, 73.35, 64.65, 62.10, 52.75, 52.69, 51.91, 47.89, 44.23, 36.36, 35.31, 34.08, 33.68, 33.14, 33.07, 31.79, 30.94, 30.50, 29.58, 28.39, 28.27, 27.74, 23.17, 23.04, 21.80, 21.41, 20.93, 11.94, 8.85. MS (ESI): *m*/*z* = 628 [M + H]^+^. HRMS: calcd. for C_30_H_49_NO_5_ [M + H]^+^: 628.2493: found: 628.2500.

#### 3.2.17. 3β-Acetoxy-12β-benzoyloxy-16β-((2-methyl-3,4-dihydro-2H-pyrrol-5-yl)oxy)-17β-(1-iodoethyl)-5α-androst-14-ene (**15**)

^1^H NMR (400 MHz, CDCl_3_) δ 8.10–8.00 (m, 2H), 7.65–7.51 (m, 1H), 7.45 (t, *J* = 7.7 Hz, 2H), 5.77–5.63 (m, 2H), 5.52 (t, *J* = 2.4 Hz, 1H), 4.88 (dd, *J* = 11.4, 4.4 Hz, 1H), 4.68 (tt, *J* = 10.8, 4.8 Hz, 1H), 4.32–4.22 (m, 1H), 3.17–2.98 (m, 1H), 2.20 (d, *J* = 0.9 Hz, 3H), 2.04–1.90 (m, 4H), 1.85 (dd, *J* = 12.5, 4.4 Hz, 2H), 1.73 (s, 2H), 1.57 (s, 8H), 1.44–1.12 (m, 11H), 1.07 (td, *J* = 13.6, 3.7 Hz, 2H), 0.96–0.81 (m, 4H). MS (ESI): ^13^C NMR (100 MHz, CDCl_3_) δ 177.58, 172.27, 170.64, 158.91, 150.34, 136.18, 129.67, 128.40, 126.29, 76.68, 73.30, 61.58, 61.58, 55.91, 52.03, 50.36, 48.31, 45.32, 43.88, 36.51, 35.58, 34.35, 33.69, 30.34, 29.69, 28.00, 27.67, 27.15, 21.89, 21.42, 16.15, 11.85. MS (ESI): *m*/*z* = 688.2 [M + H]^+^.

#### 3.2.18. 3β-Acetoxy-16β-((2-methyl-3,4-dihydro-2H-pyrrol-5-yl)oxy)-17β-(1-iodoethyl)-5α-androstane (**18**)

^1^H NMR (400 MHz, CDCl_3_) δ 4.97 (tt, *J* = 10.8, 4.8 Hz, 1H), 4.55 (dd, *J* = 11.2, 4.6 Hz, 1H), 4.29 (t, *J* = 6.6 Hz, 1H), 3.35–3.23 (m, 2H), 2.10–1.95 (m, 5H), 1.84–1.43 (m, 10H), 1.41–1.11 (m, 14H), 1.04–0.79 (m, 10H). ^13^C NMR (100 MHz, CDCl_3_) δ 177.56, 172.26, 170.63, 82.38, 79.98, 73.35, 63.46, 62.18, 52.72, 51.49, 51.01, 47.78, 45.26, 37.42, 35.61, 34.77, 33.92, 33.26, 32.56, 31.78, 30.87, 30.51, 29.67, 28.97, 27.73, 23.80, 23.09, 22.81, 21.42, 14.35, 12.22. MS (ESI): *m*/*z* = 570.2 [M + H]^+^.

## 4. Conclusions

We have presented the first example of the hypervalent iodine-mediated formation of spiroiminals that occur, presumably, via tandem aminyl radical cyclization and amine-to-imine oxidation. To the best of our knowledge, this reaction represents the first reported instance of C-H activation-mediated spiroiminal formation. The substitution at the Cα position of the amine greatly impacts the hypervalent iodine-mediated cyclization of steroidal amines, resulting in varying product distributions of spiroiminals, spiroaminals, and oxyimines. Synthetic efforts to utilize this novel radical cyclization are underway and the results will be reported elsewhere in due course.

## Figures and Tables

**Table 1:** Optimization of 5/5-spiroiminal formation.

Entry	Solvents	Molar Equiv. of PhI(OAc)_2_/I_2_	Time (h)	Product Distribution		
				Spiroiminal 10 (%)	Spiroaminal 11 (%)	Iodo 12 (%)
1	CH_3_CN	2/2	2	32	9	25
2	CH_3_CN	4/4	1	54	8	12
3	CH_3_CN	6/6	0.5	65	11	9
4	CH_2_Cl_2_	6/6	0.5	31	18	20
5	THF	6/6	0.5	21	16	42
6	Dioxane ^a^	6/6	1	ND	ND	ND
7	Benzene ^b^	6/6	1	38	15	7

Note: ^a^ This reaction was conducted at 25 °C and 62% of the starting material was recovered. No reaction occurred, even at 25 °C. ^b^ A total of 11% of the starting material was recovered from the reaction. ND: not detected.

**Table 2:** PhI(OAc)_2_/I_2_-mediated cyclization of steroidal amines (**8**) and (**9**).

Entry	Substrate	Products
1	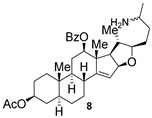	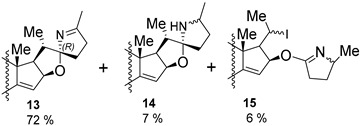
2	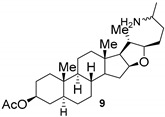	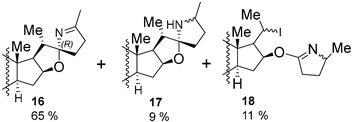

Note: Unless otherwise noted, all reactions were carried out with PhI(OAc)_2_ (6 equiv.) and iodine (6 equiv.) in CH_3_CN (0.1 M) at 0 °C. A steroidal amine (1 equiv.) was added and the resulting mixture was stirred at 0 °C. The yield and reaction time of the isolated products aregiven.

## Data Availability

Data are contained within the article and Supplementary Materials.

## Supplementary Materials

The following supporting information can be downloaded at: https://www.mdpi.com/1420-3049/29/23/5812/s1, Figure S1: View of 10 showing the atom labeling scheme; Scheme S1: Complete synthesis for spiroiminal 10 formation from hecogenin acetate; Scheme S2: Complete synthesis for spiroiminal 13 formation from hecogenin acetate; Scheme S3: Complete synthesis for spiroiminal 16 formation from diosgenin acetate; Table S1: Crystal data and structure refinement for 10; Table S2: Atomic coordinates (×10^4^) and equivalent isotropic displacement parameters (Å^2^ × 10^3^) for 10; Table S3: Bond lengths [Å] and angles [°] for 10; Table S4: Anisotropic displacement parameters (Å^2^ × 10^3^) for 10; Table S5: Hydrogen coordinates (×10^4^) and isotropic displacement parameters (Å^2^ × 10^3^) for 10; Table S6: Torsion angles [°] for 10.
