# Supplementary material for: Hypervalent Iodine-Mediated Synthesis of Steroidal 5/5-Spiroiminals
Source: Molecules. 2024 Dec 9;29(23):5812. doi: 10.3390/molecules29235812 (PMC12068023; doi:10.3390/molecules29235812)
Supplement: Supplementary file 1 [file molecules-29-05812-s001.zip › molecules-3257481-supplementary.pdf]

Supporting Information

Hypervalent iodine-mediated synthesis of 5/5-spiroiminals

Rayala Naveen Kumar and Seongmin Lee*

The Division of Chemical Biology and Medicinal Chemistry, College of Pharmacy,
University of Texas at Austin, Austin, TX 78712, USA

*Corresponding Author. Tel.: +1-512-471-1785; Fax: +1-512-471-4726.
E-mail address: SeongminLee@austin.utexas.edu (Seongmin Lee)

1. Synthesis Schemes and ^{1}H NMR and ^{13}C NMR Spectra of All New Compounds S2 – S19

2. Crystal Data of **10** S20 – S33

Experimental Section

All reactions were performed under positive pressure of argon in anhydrous solvents. Each reaction progress was monitored by thin layer chromatography (TLC). TLC Silica gel 60 F254 glass plates from EMD Chemicals Inc. (Darmstadt, Germany) and appropriate solvent systems were used for TLC development. TLC plates were visualized by ultraviolet illumination (254 nm) and *p*-Anisaldehyde solution (4 mL of concentrated sulfuric acid, 800 mL of ethanol, 1.2 mL of acetic acid, and 1.6 mL of p-anisaldehyde). Analytical samples were prepared via flash silica gel chromatography. 60 Å silica from Bonna-Agela technologies (Wilmington, DE) was used to purify the products. 1H and ^{13}C NMR spectra were generated by Varian Mercury 400 MHz spectrometer. $CDCl_3$ was used as the NMR standard. Peak multiplicities in ^1H NMR spectra, when reported, were abbreviated as s (singlet), d (doublet), t (triplet), m (multiplet), ap (apparent), and br (broad). High-resolution mass spectrometry data were generated by Agilent 6530 Accurate-Mass Q-TOF LC/MS.

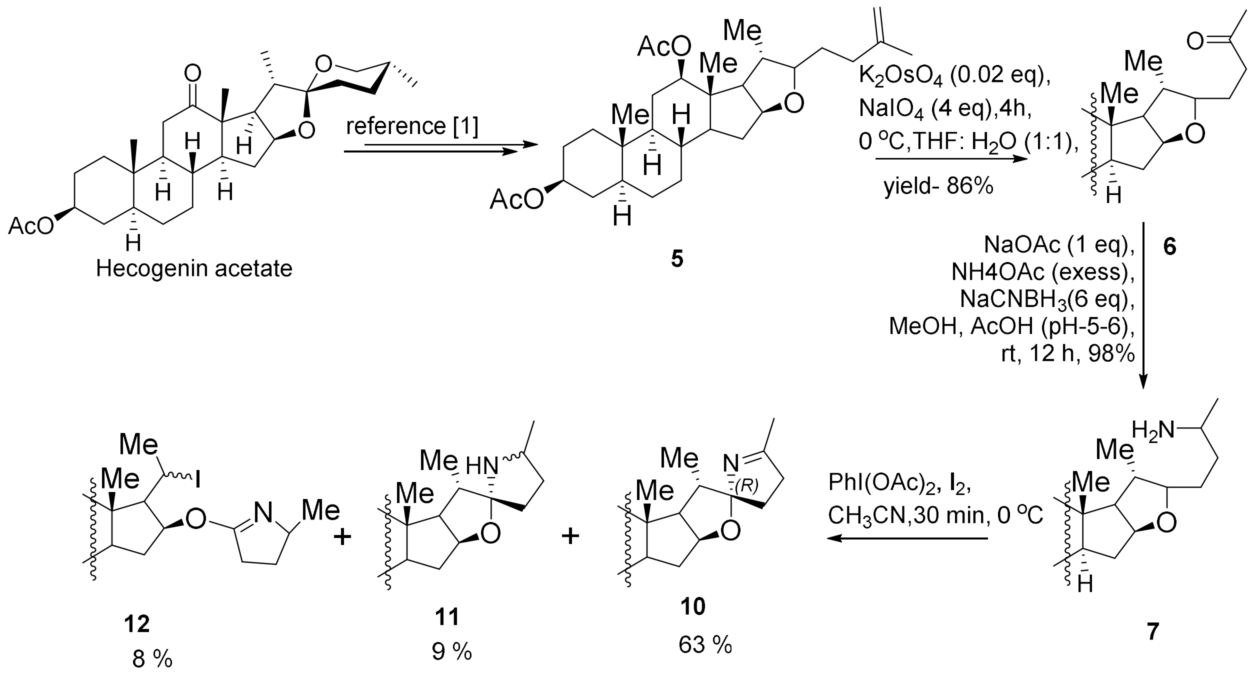

Scheme S1 Complete synthesis for spiroiminal **10** formation from hecogenin acetate**.**

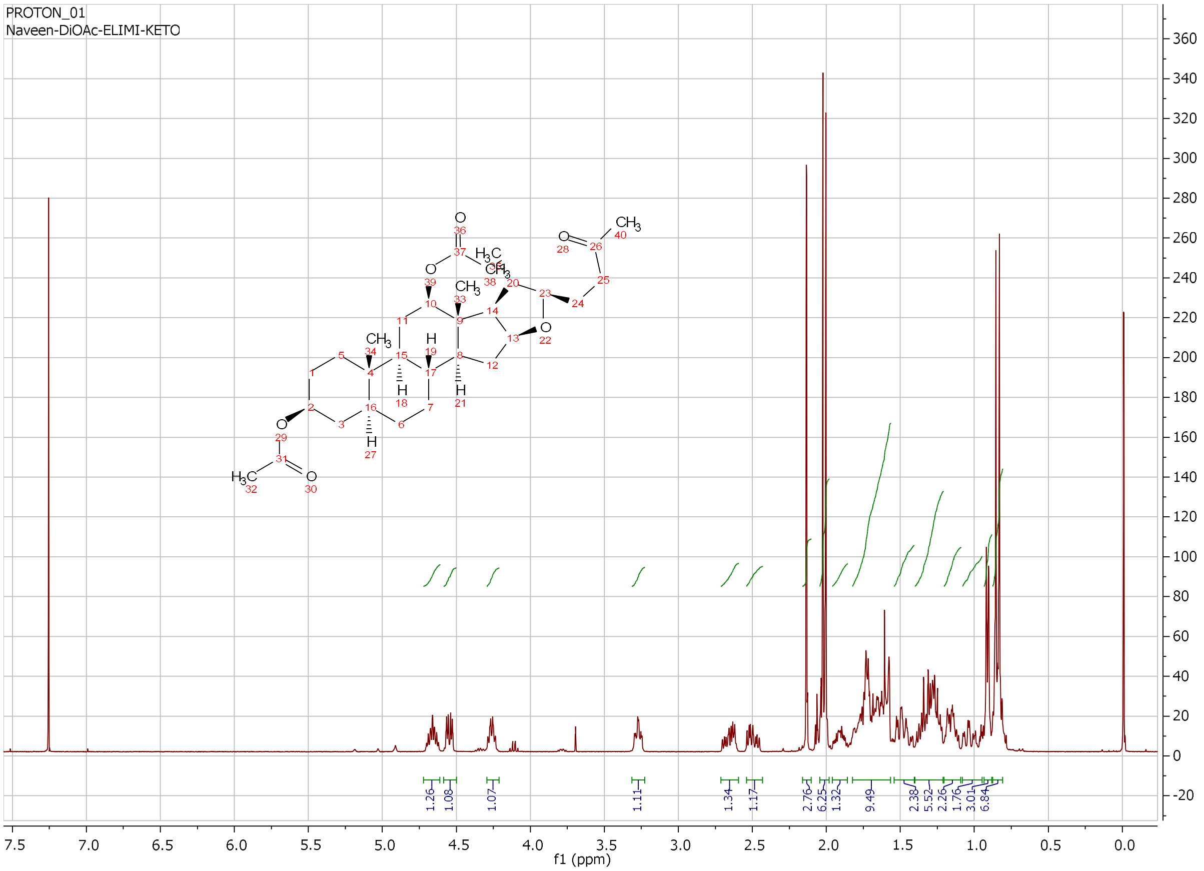
PROTON_01
Naveen-DiOAc-ELIMI-KETO
1.26
1.08
1.07
1.11
1.34
1.17
2.76
6.25
1.32
9.49
2.38
5.52
2.26
1.76
3.01
6.84
f1 (ppm)

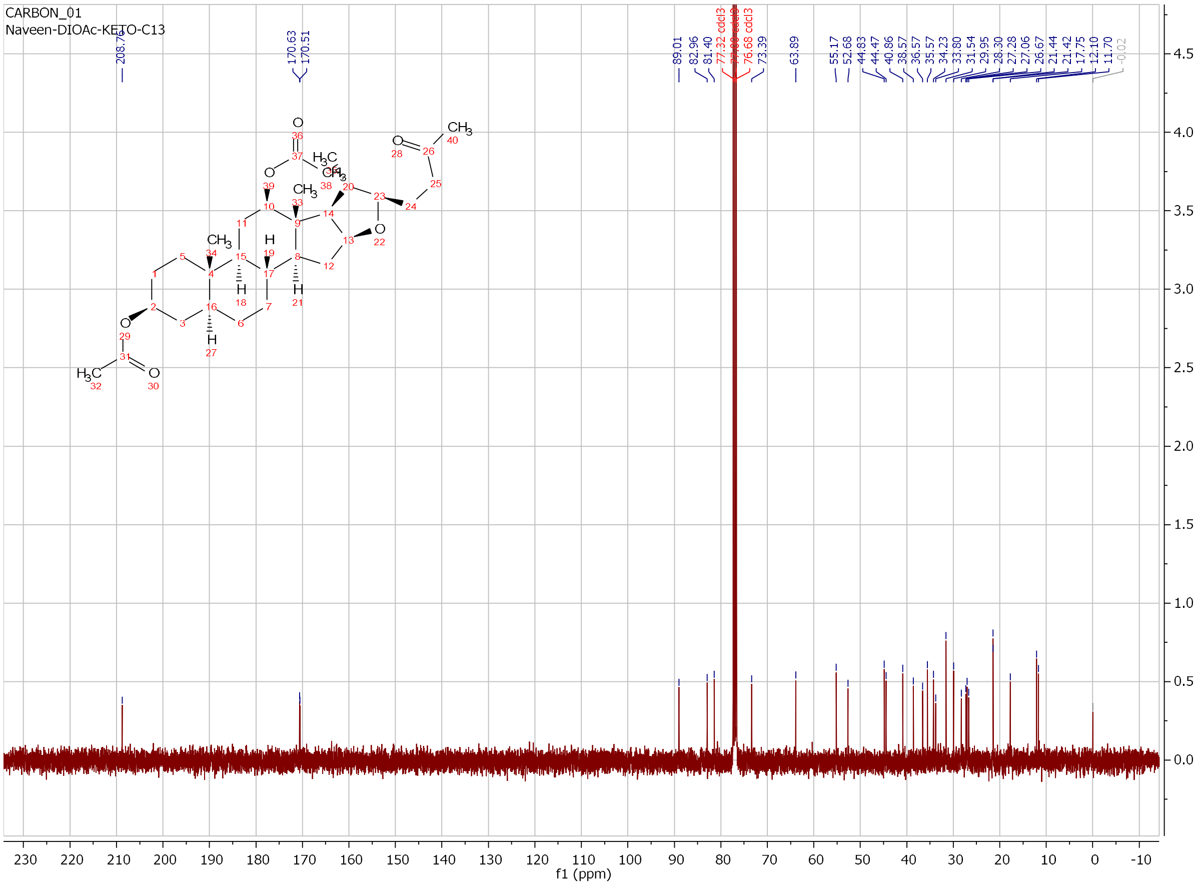
CARBON_01
Naveen-DIOAc-KETO-C13
208.76
170.63
170.51
89.01
82.96
81.40
77.32 cdcl3
76.68 cdcl3
73.39
63.89
55.17
52.68
44.83
44.47
40.86
38.57
36.57
35.57
34.23
33.80
31.54
29.95
28.30
27.28
27.06
26.67
21.44
21.42
17.75
12.10
11.70
-0.02
f1 (ppm)

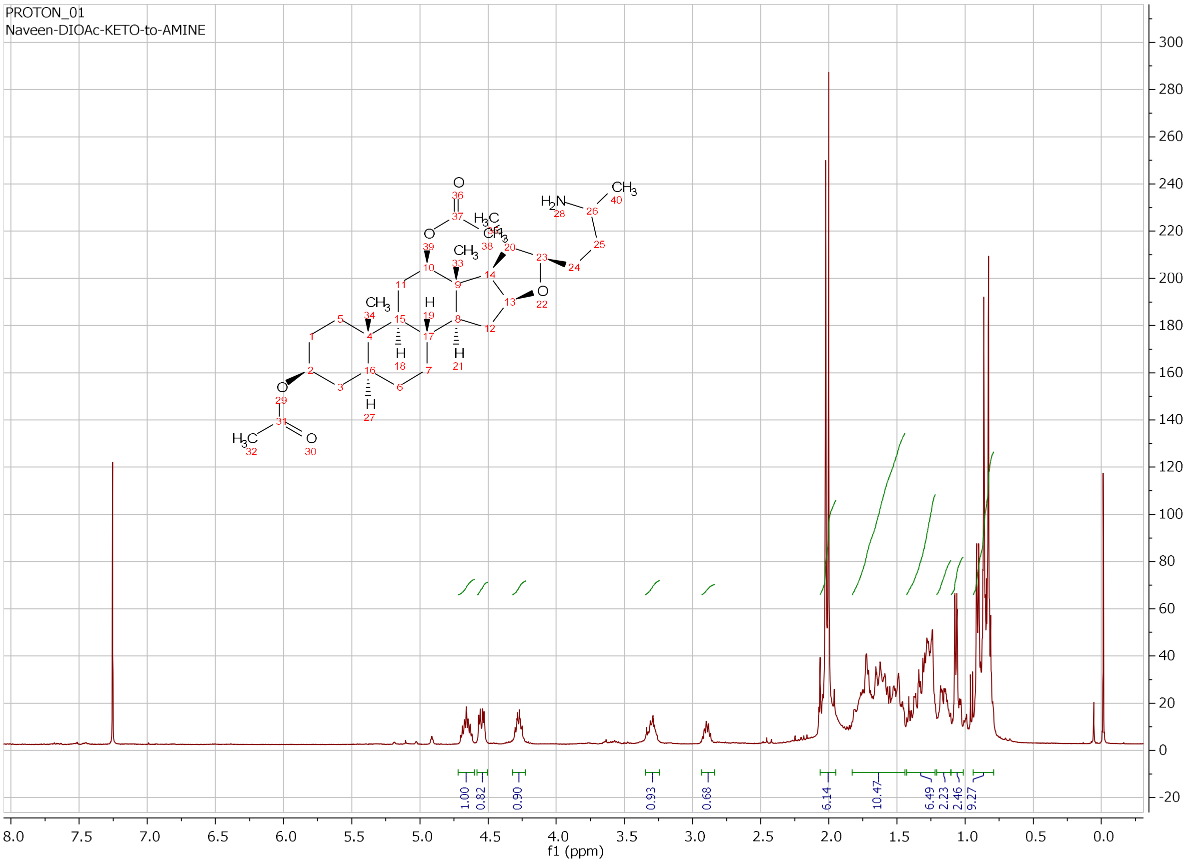
PROTON_01
Naveen-DIOAc-KETO-to-AMINE
1.00
0.82
0.90
0.93
0.68
6.14
10.47
6.49
2.23
2.46
9.27
f1 (ppm)

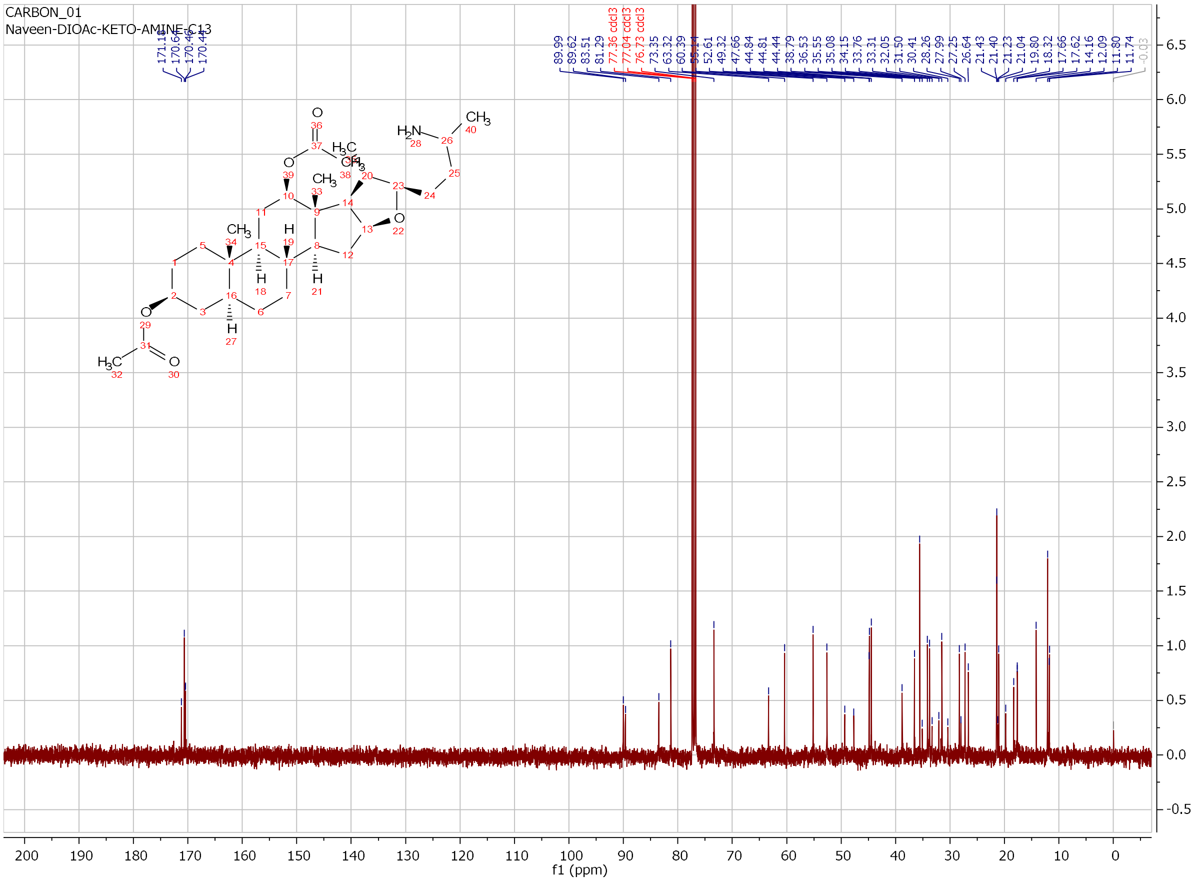
CARBON_01
Naveen-DIOAc-KETO-AMINE-C13
171.18
170.64
170.46
170.44
89.99
89.62
83.51
81.29
77.36 cdcl3
77.04 cdcl3
76.73 cdcl3
73.35
63.32
60.39
52.61
49.32
47.66
44.84
44.81
44.44
38.79
36.53
35.55
35.08
34.15
33.76
33.31
32.05
31.50
30.41
28.26
27.99
27.25
26.64
21.43
21.40
21.23
21.04
19.80
18.32
17.66
17.62
14.16
12.09
11.80
11.74
-0.03
f1 (ppm)

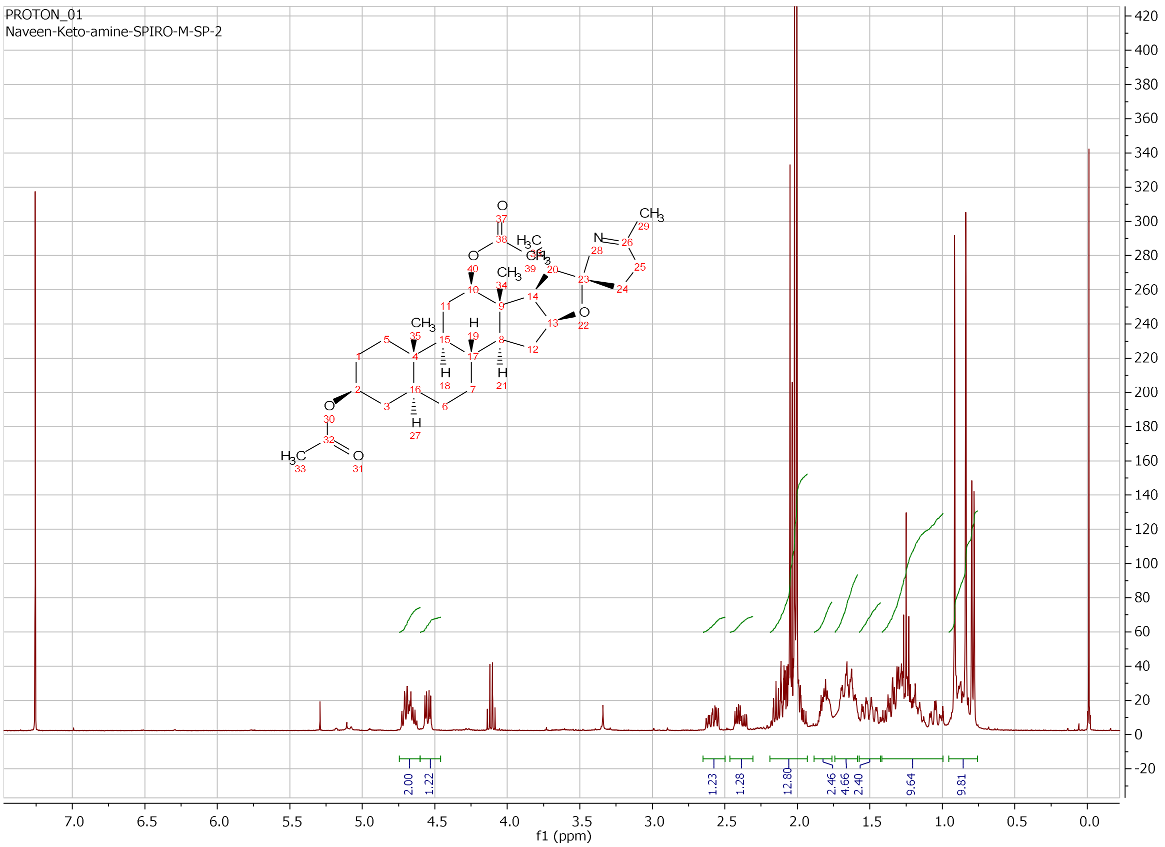

PROTON_01
Naveen-Keto-amine-SPIRO-M-SP-2
2.00
1.22
1.23
1.28
12.80
2.46
4.66
2.40
9.64
9.81
f1 (ppm)

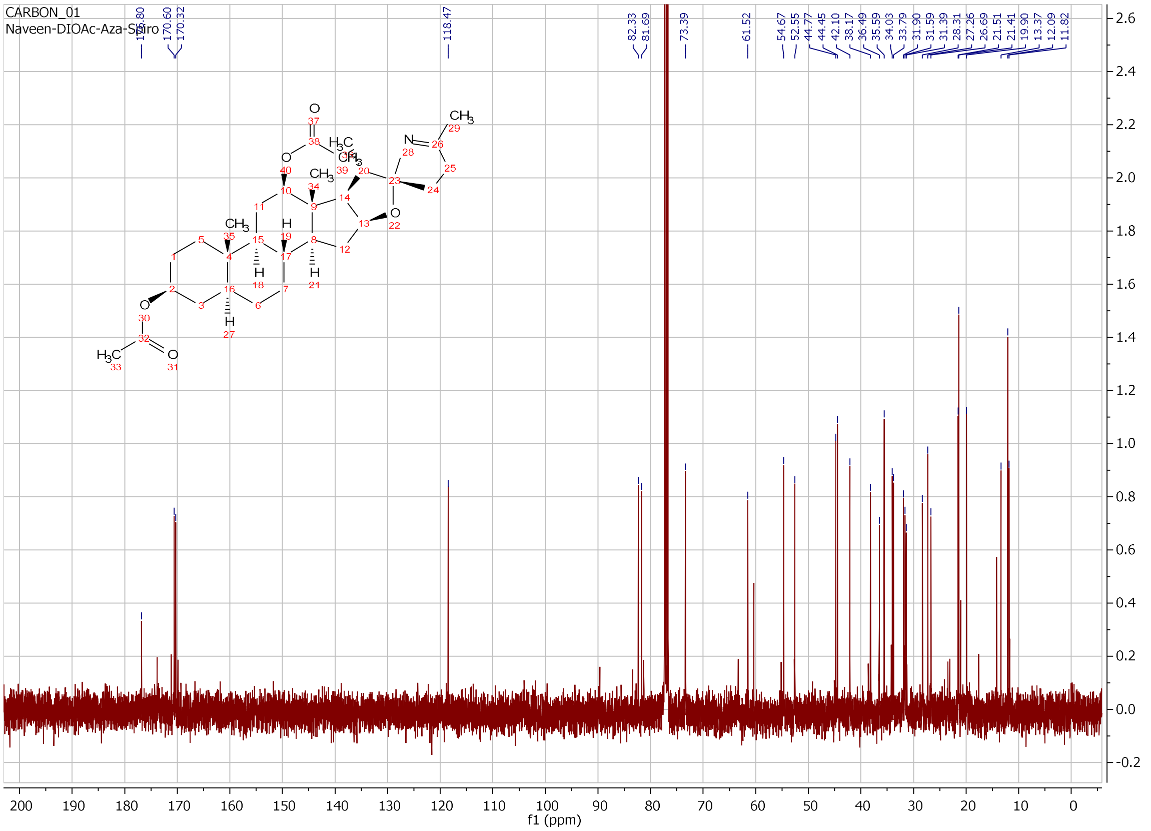

CARBON_01
Naveen-DIOAc-Aza-Spiro
170.60
170.32
118.47
82.33
81.69
73.39
61.52
54.67
52.55
44.77
44.45
42.10
38.17
36.49
35.59
34.03
33.79
31.90
31.59
31.39
28.31
27.26
26.69
21.51
21.41
19.90
13.37
12.09
11.82
f1 (ppm)

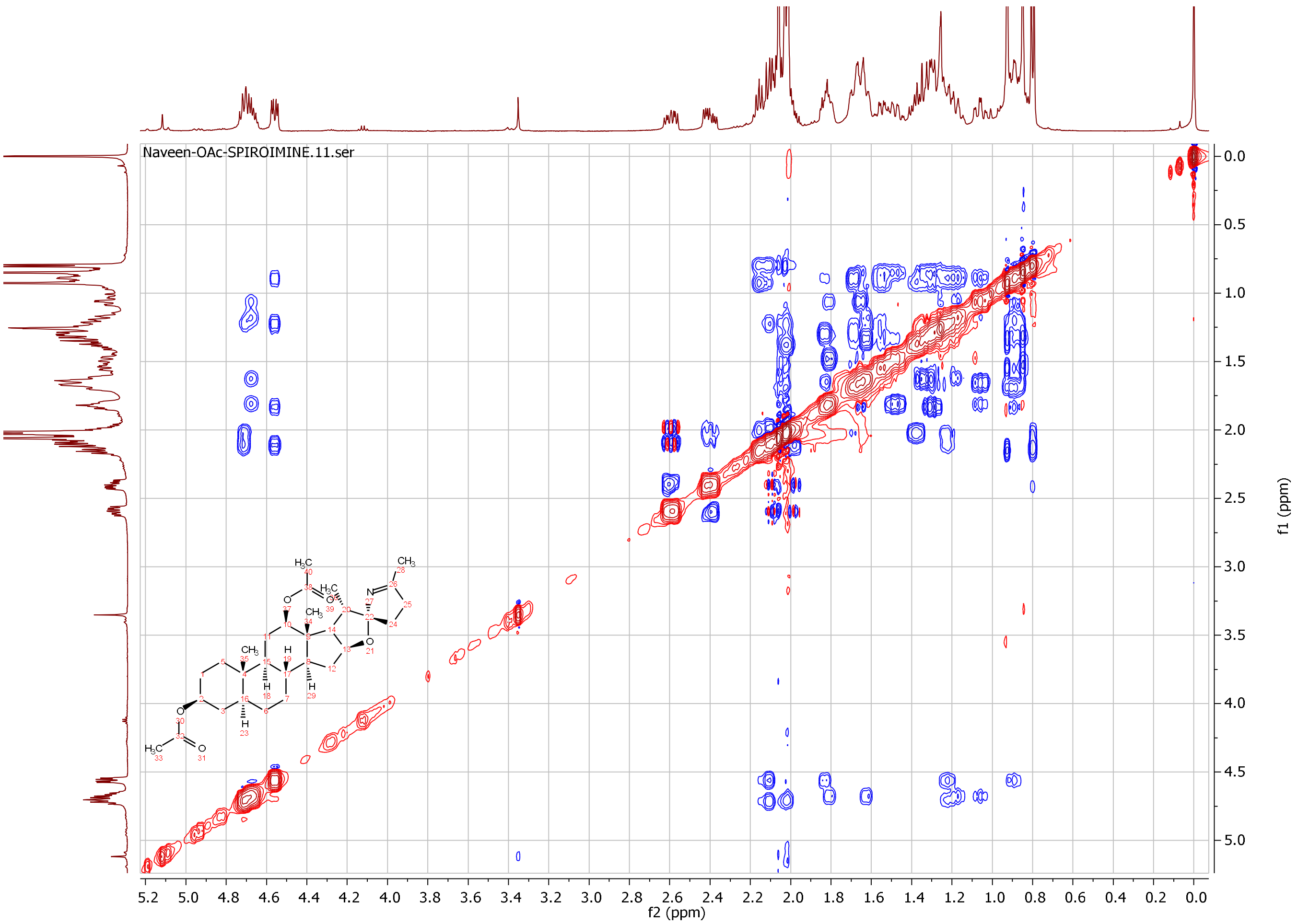
Naveen-OAc-SPIROIMINE.11.ser
f2 (ppm)
f1 (ppm)

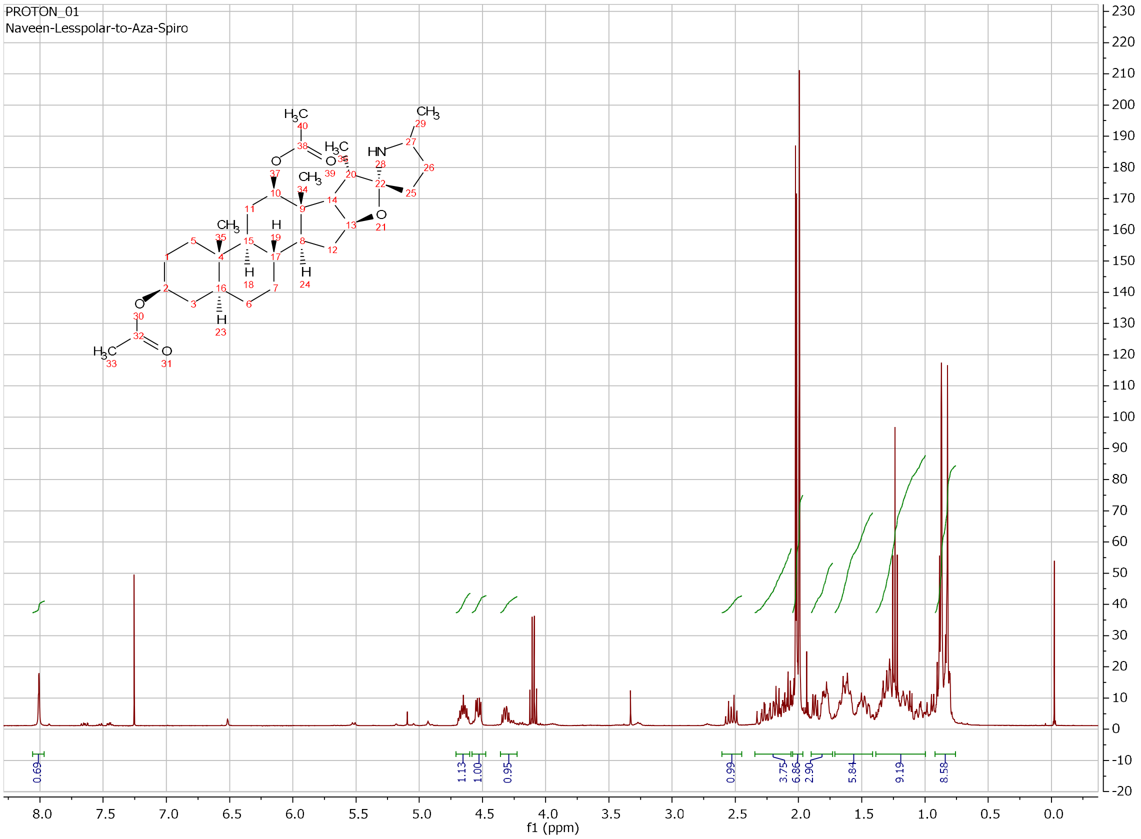
PROTON_01
Naveen-Lesspolar-to-Aza-Spiro
0.69
1.13
1.00
0.95
0.99
3.75
6.86
2.90
5.84
9.19
8.58
f1 (ppm)

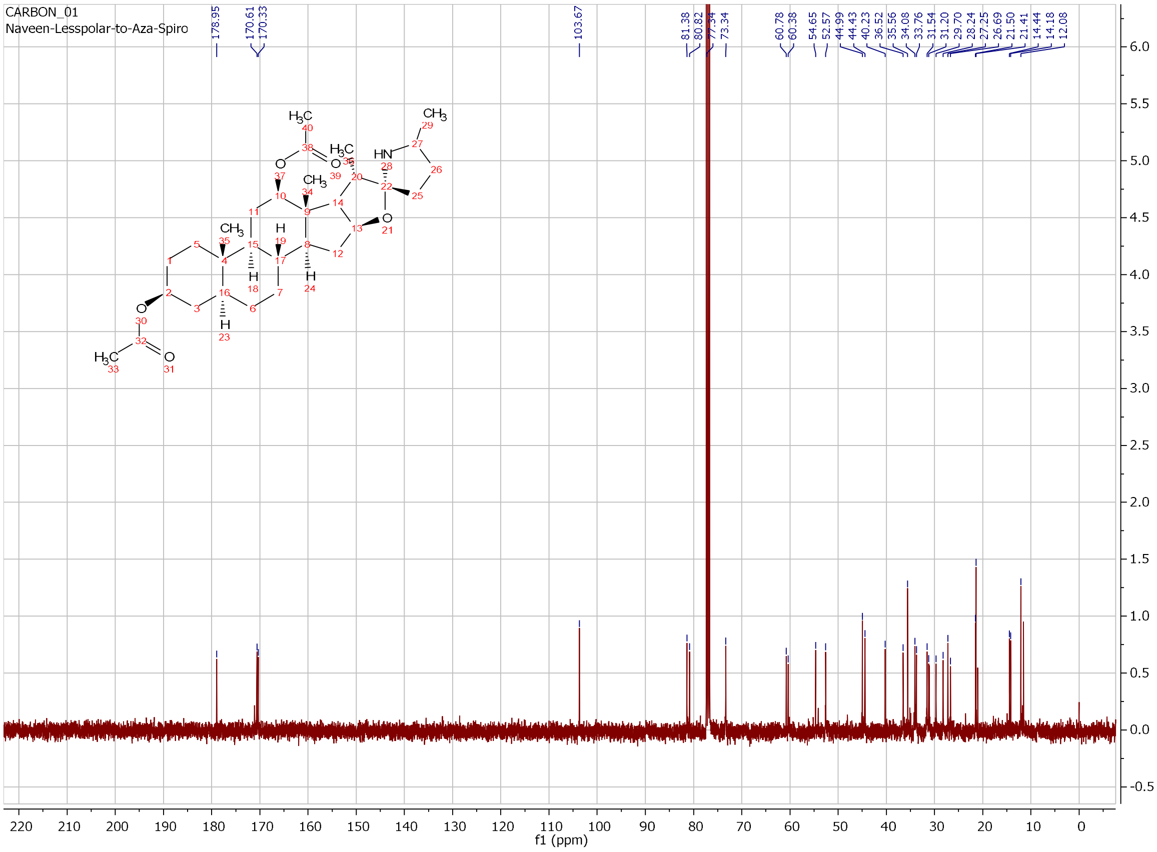
CARBON_01
Naveen-Lesspolar-to-Aza-Spiro
178.95
170.61
170.33
103.67
81.38
80.82
77.34
73.34
60.78
60.38
54.65
52.57
44.99
44.43
40.23
36.52
35.56
34.08
33.76
31.54
31.20
29.70
28.24
27.25
26.69
21.50
21.41
14.44
14.18
12.08
f1 (ppm)

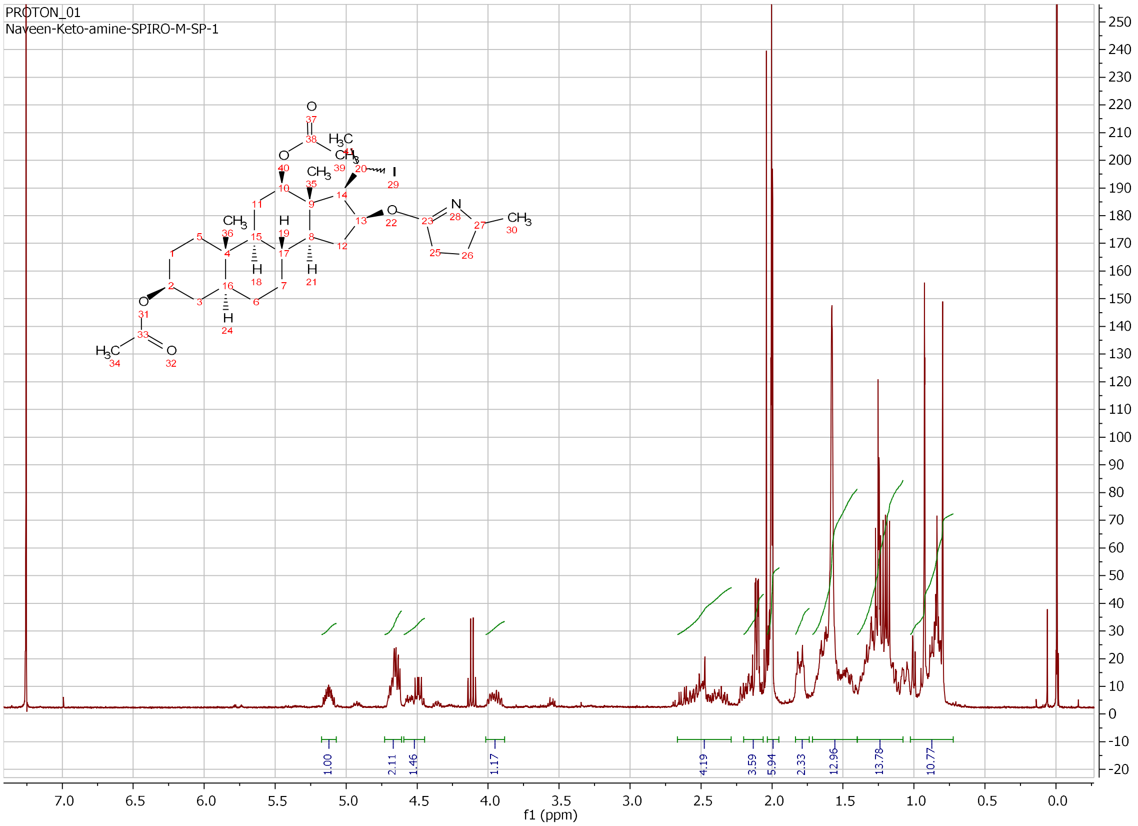

PROTON_01
Naveen-Keto-amine-SPIRO-M-SP-1
1.00
2.11
1.46
1.17
4.19
3.59
5.94
2.33
12.96
13.78
10.77
f1 (ppm)

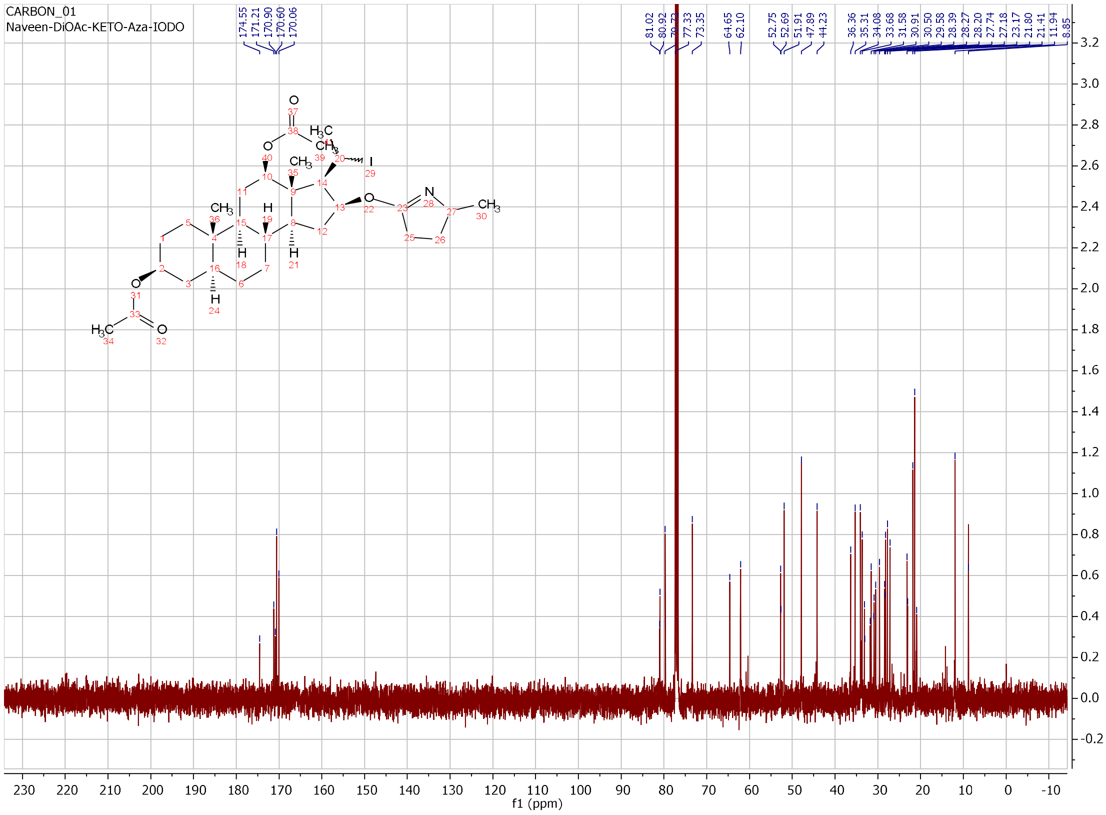

CARBON_01
Naveen-DiOAc-KETO-Aza-IODO
174.55
171.21
170.90
170.60
170.06
81.02
80.92
77.33
73.35
64.65
62.10
52.75
52.69
51.91
47.89
44.23
36.36
35.31
34.08
33.68
31.58
30.91
30.50
29.58
28.39
28.27
28.20
27.74
27.18
23.17
21.80
21.41
11.94
8.85
f1 (ppm)

Scheme S2 Complete synthesis for spiroiminal **13** formation from hecogenin acetate**.**

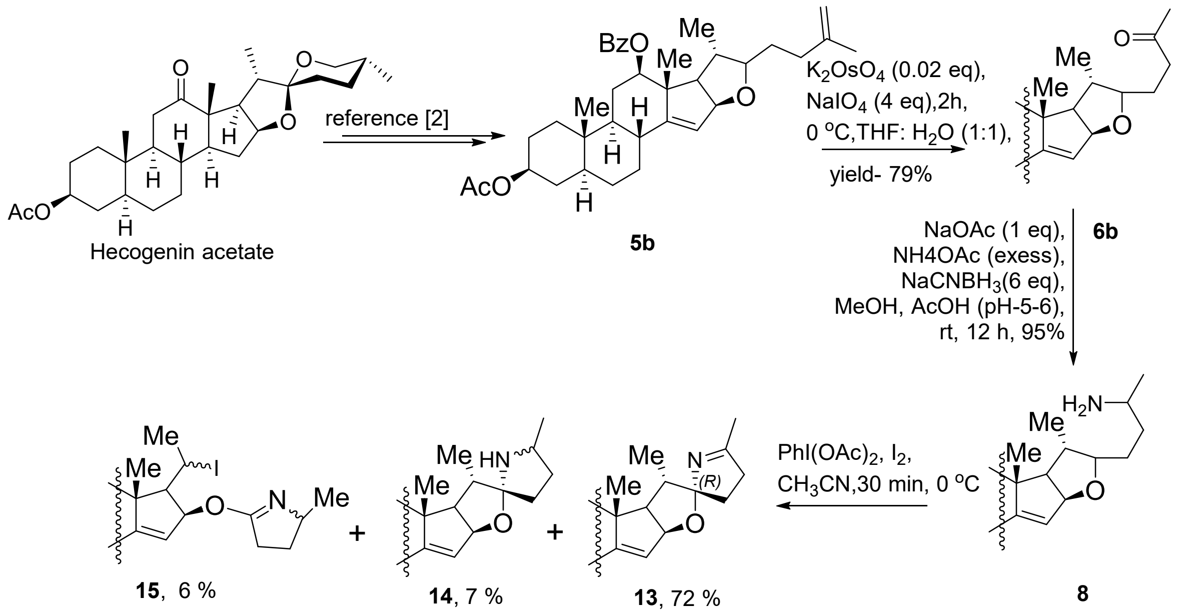

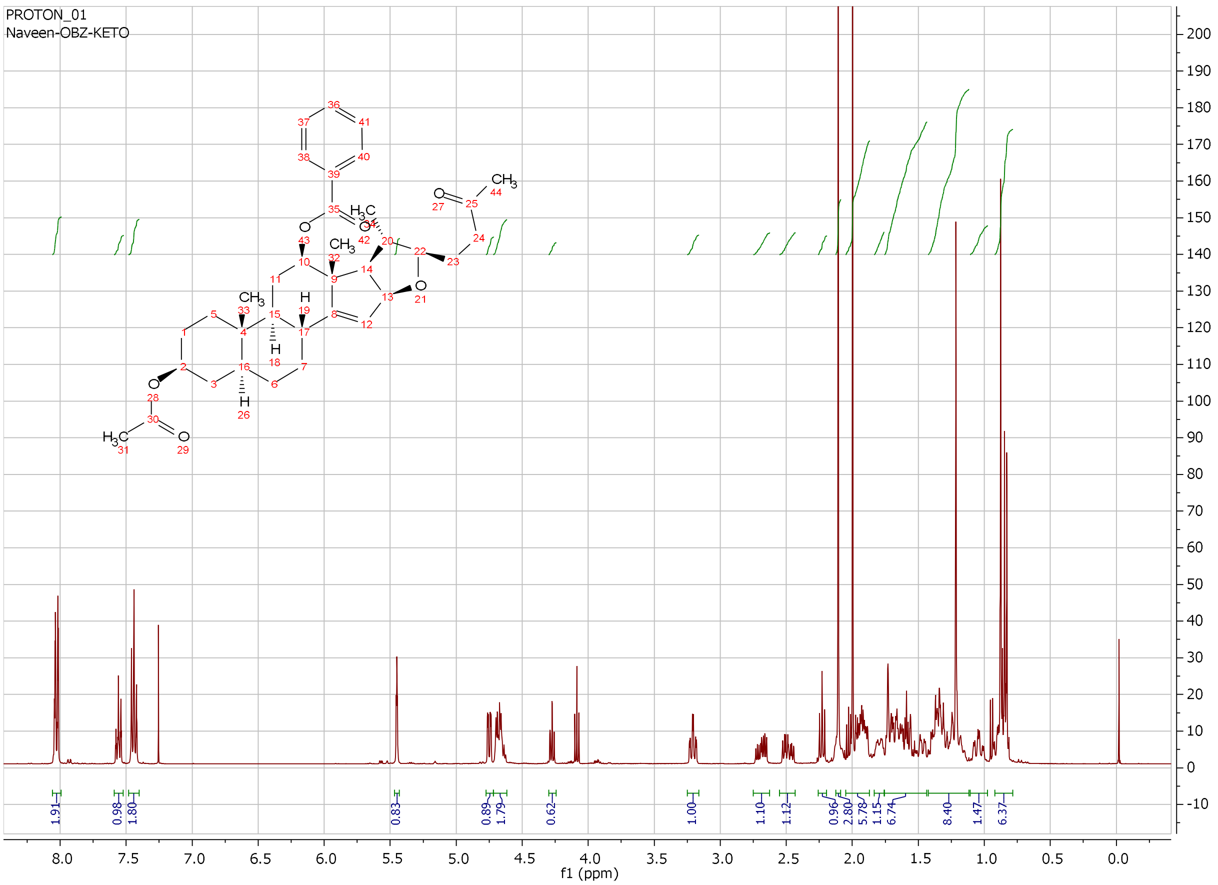
PROTON_01
Naveen-OBZ-KETO
1.91
0.98
1.80
0.83
0.89
1.79
0.62
1.00
1.10
1.12
0.96
2.80
5.78
1.15
6.74
8.40
1.47
6.37
f1 (ppm)

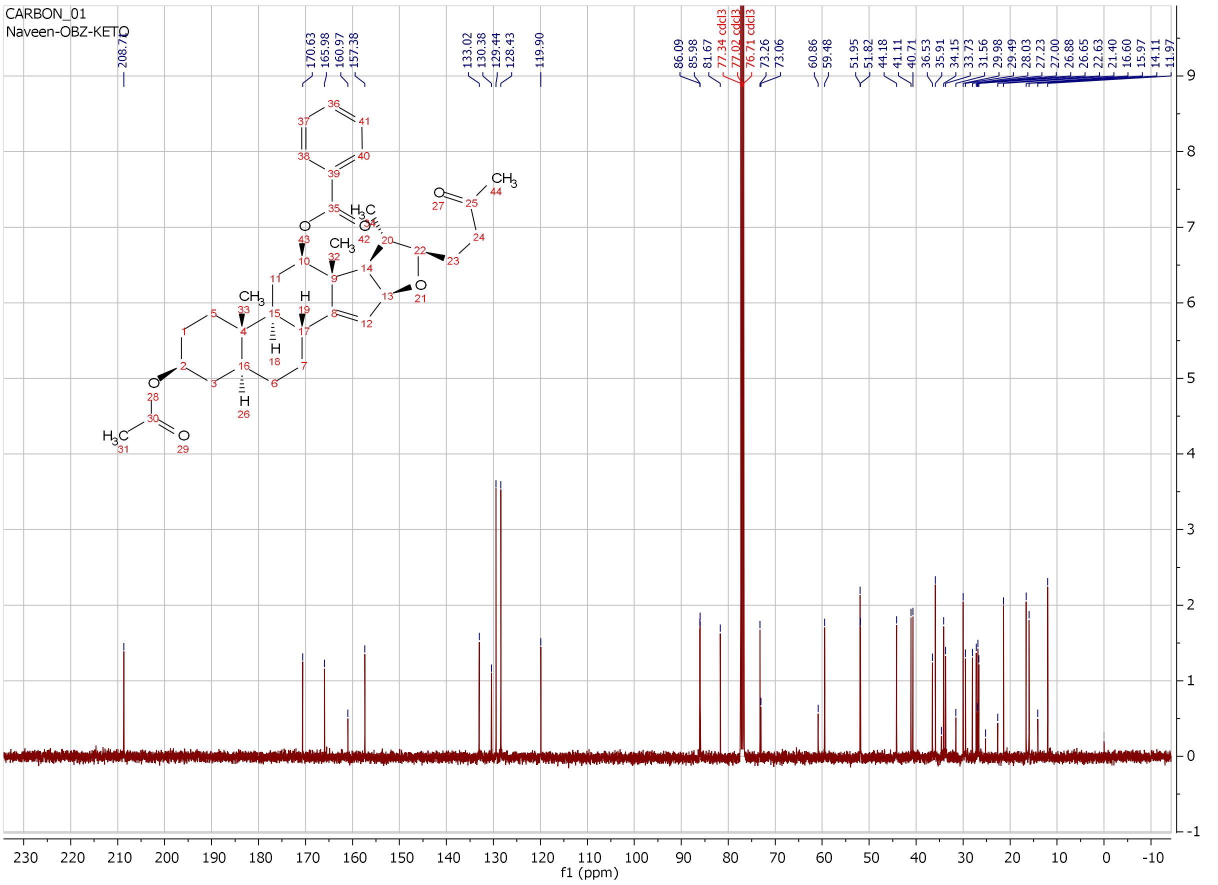
CARBON_01
Naveen-OBZ-KETO
208.70
170.63
165.98
160.97
157.38
133.02
130.38
129.44
128.43
119.90
86.09
85.98
81.67
77.34 cdcl3
77.02 cdcl3
76.71 cdcl3
73.26
73.06
60.86
59.48
51.95
51.82
44.18
41.11
40.71
36.53
35.91
34.15
33.73
31.56
29.98
29.49
28.03
27.23
27.00
26.88
26.65
22.63
21.40
16.60
15.97
14.11
11.97
f1 (ppm)

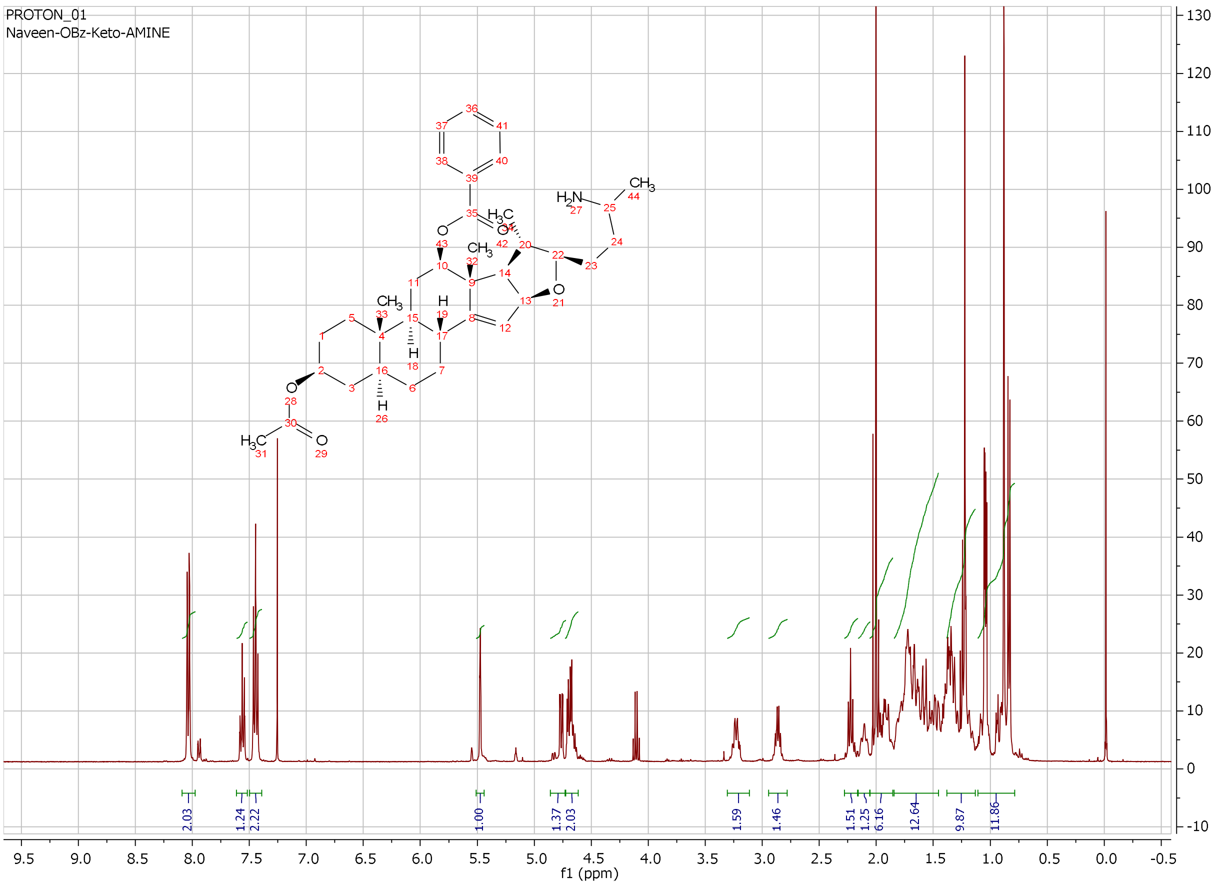

PROTON_01
Naveen-OBz-Keto-AMINE
2.03
1.24
2.22
1.00
1.37
2.03
1.59
1.46
1.51
1.25
6.16
12.64
9.87
11.86
f1 (ppm)

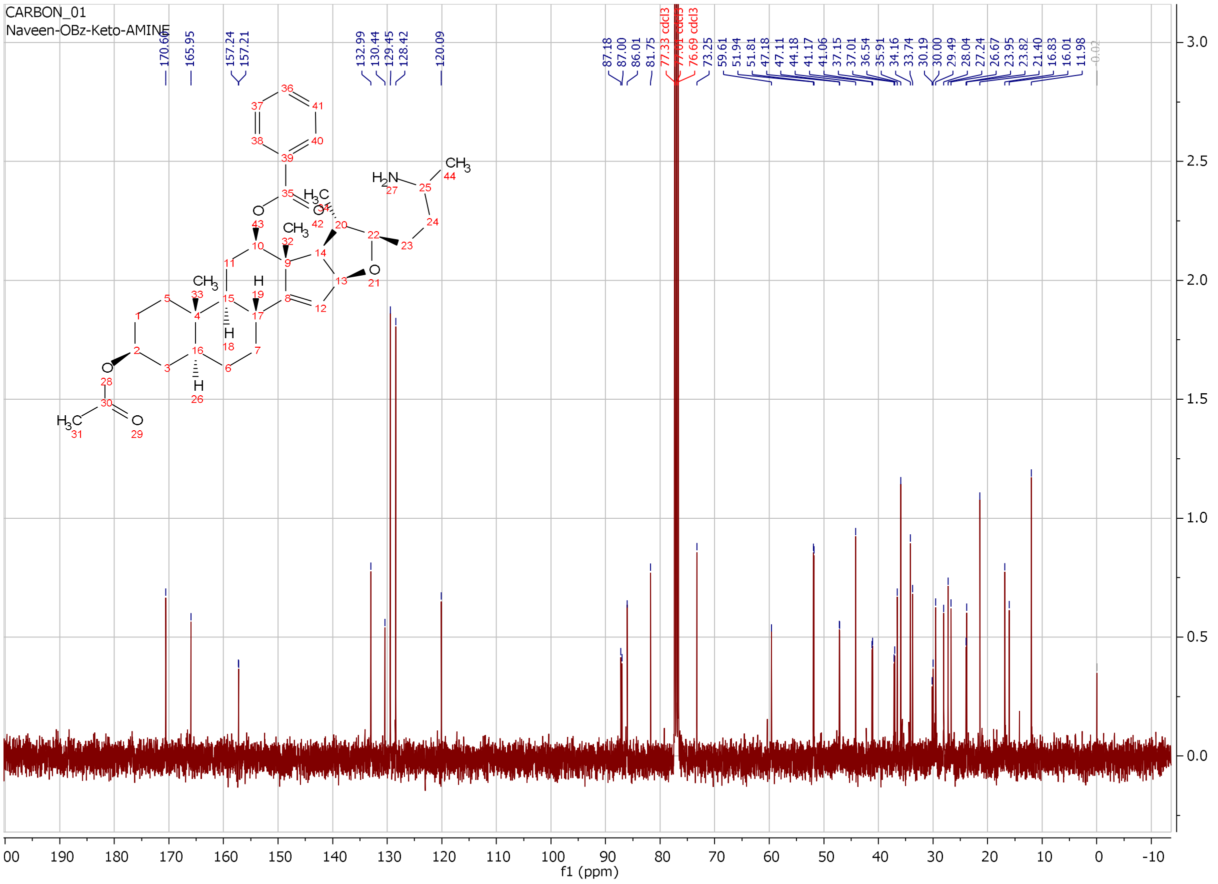

CARBON_01
Naveen-OBz-Keto-AMINE
170.66
165.95
157.24
157.21
132.99
130.44
129.45
128.42
120.09
87.18
87.00
86.01
81.75
77.33 cdcl3
77.01 cdcl3
76.69 cdcl3
73.25
59.61
51.94
51.81
47.18
47.11
44.18
41.17
41.06
37.15
37.01
36.54
35.91
34.16
33.74
30.19
30.00
29.49
28.04
27.24
26.67
23.95
23.82
21.40
16.83
16.01
11.98
-0.02
f1 (ppm)

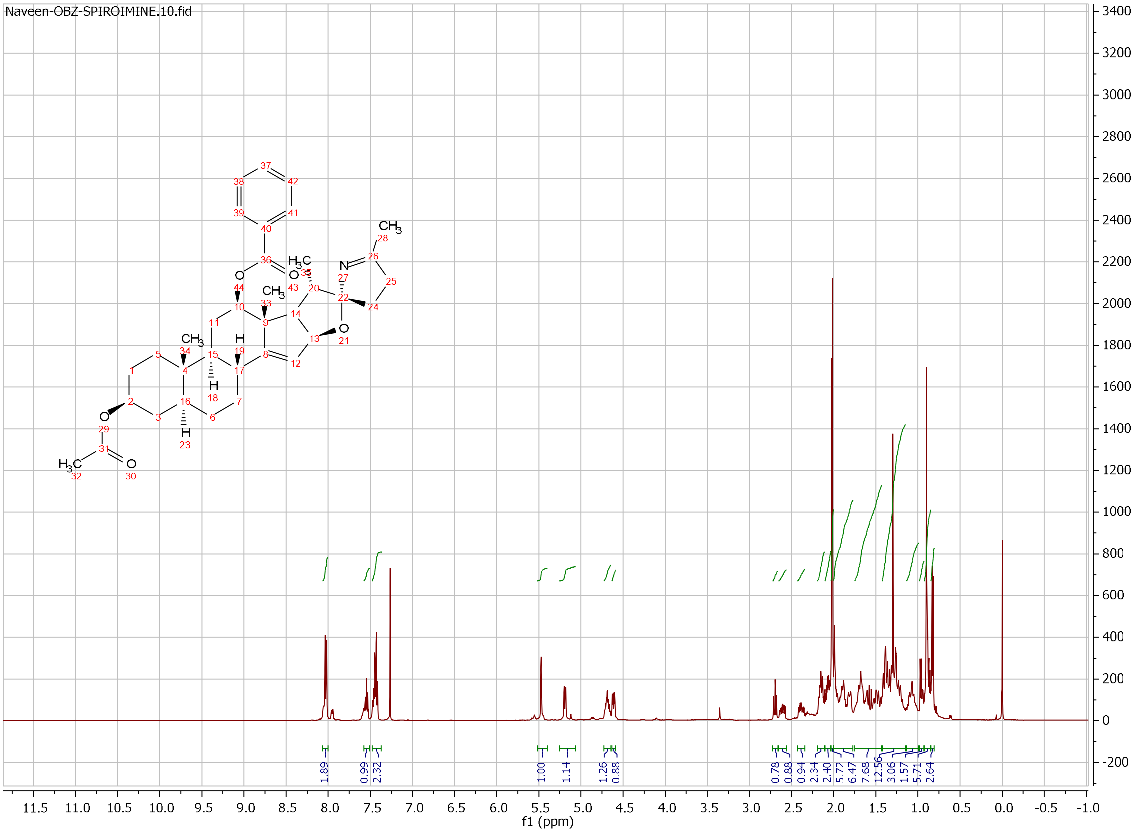
Naveen-OBZ-SPIROIMINE.10.fid
1.89
0.99
2.32
1.00
1.14
1.26
0.88
0.78
0.88
0.94
2.34
2.40
5.72
6.47
7.68
12.56
3.06
1.57
5.71
2.64
f1 (ppm)

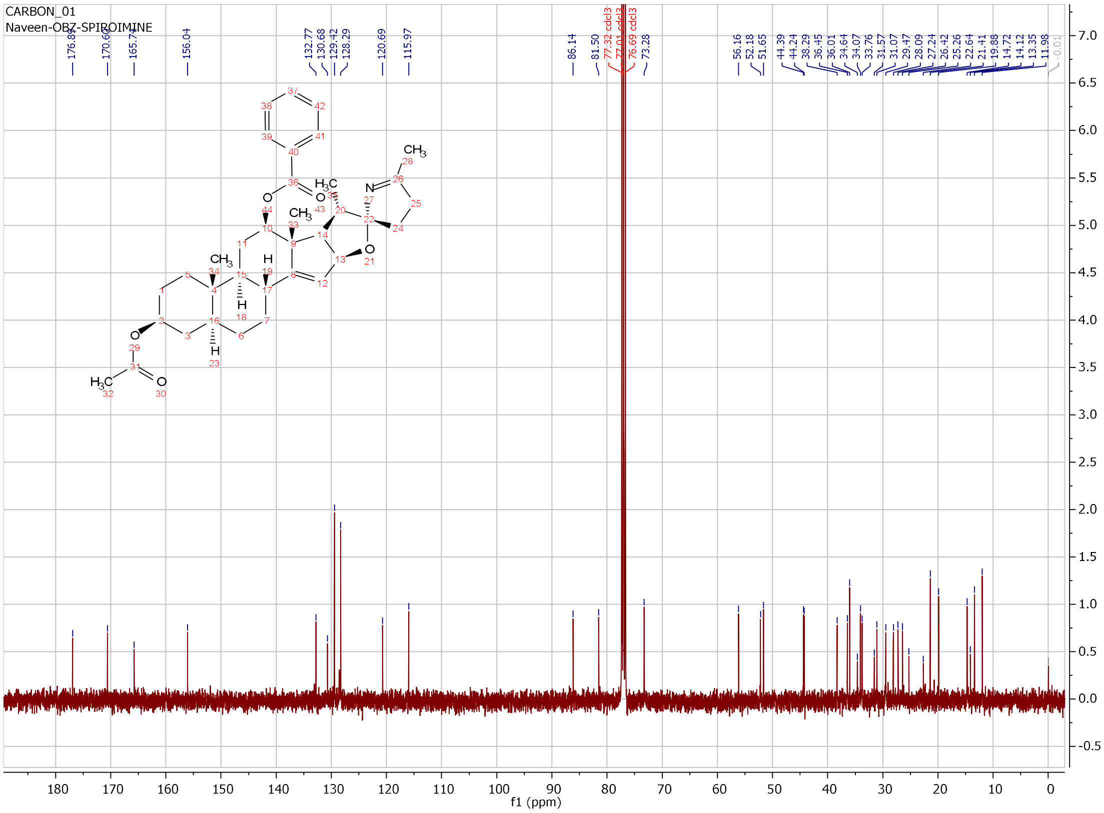
CARBON_01
Naveen-OBZ-SPIROIMINE
156.04
132.77
130.68
129.42
128.29
120.69
115.97
86.14
81.50
77.32 cdcl3
77.01 cdcl3
76.69 cdcl3
73.28
56.16
52.18
51.65
44.39
44.24
38.29
36.45
36.01
34.64
34.07
33.76
31.57
31.07
29.47
28.09
27.24
26.42
25.26
22.64
21.41
19.88
14.72
14.12
13.35
11.98
f1 (ppm)

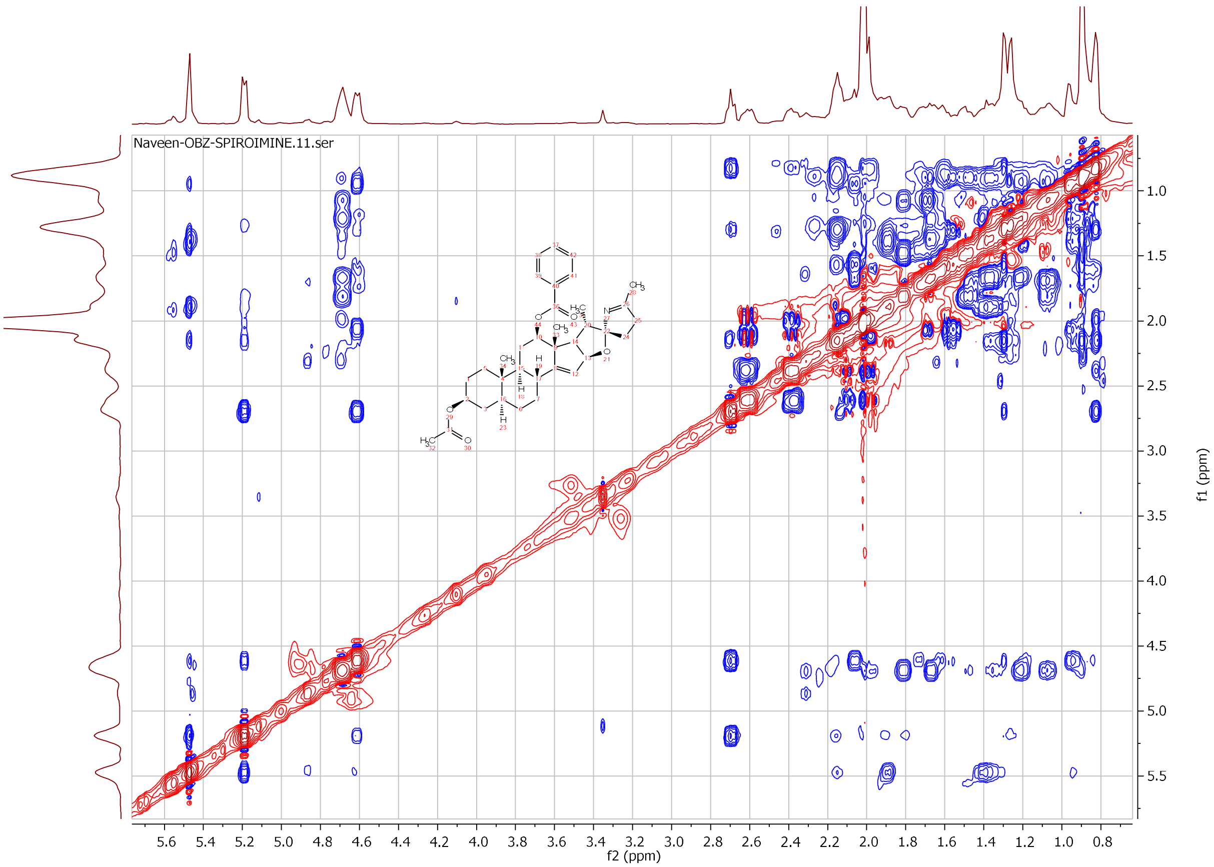
Naveen-OBZ-SPIROIMINE.11.ser
f1 (ppm)
f2 (ppm)

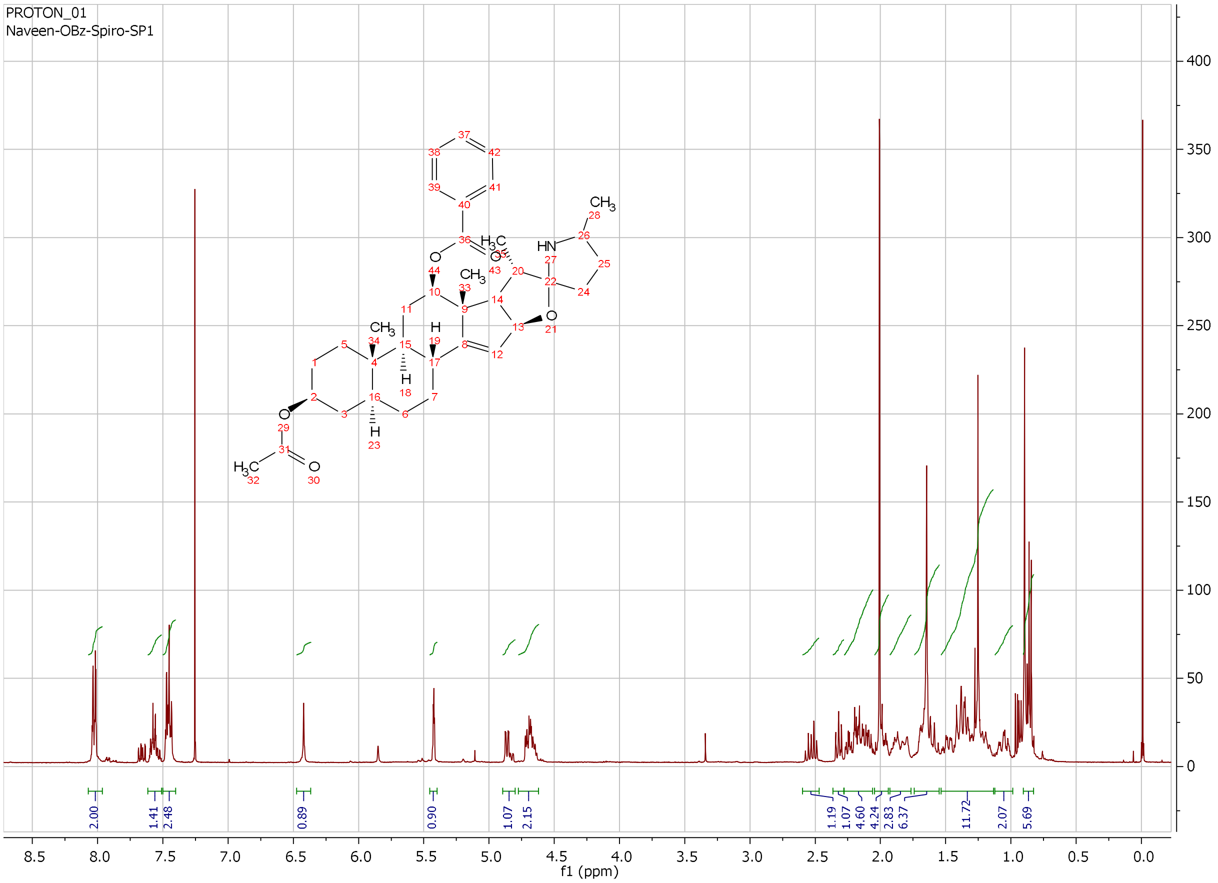
PROTON_01
Naveen-OBz-Spiro-SP1
f1 (ppm)

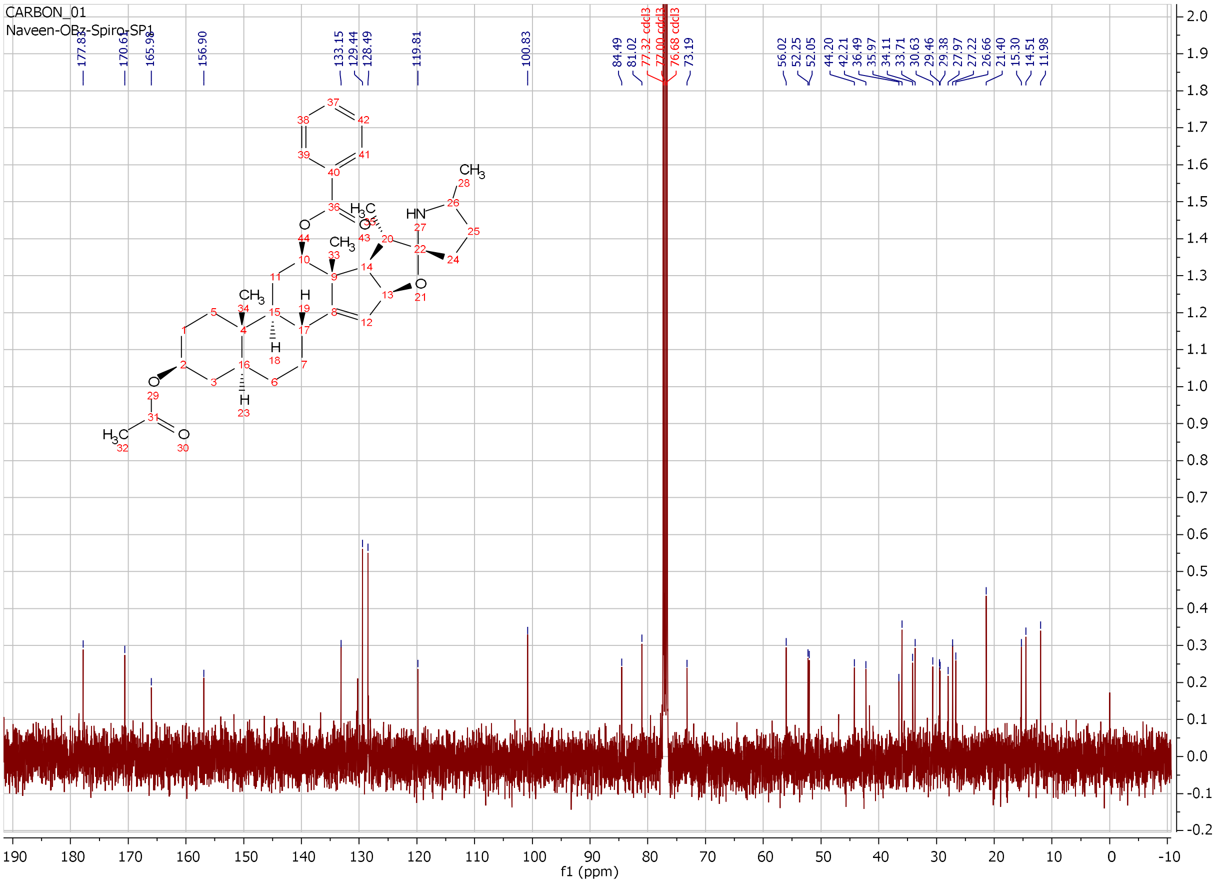
CARBON_01
Naveen-OBz-Spiro-SP1
177.83
170.61
165.98
156.90
133.15
129.44
128.49
119.81
100.83
84.49
81.02
77.32 cdcl3
77.00 cdcl3
76.68 cdcl3
73.19
56.02
52.25
52.05
44.20
42.21
36.49
35.97
34.11
33.71
30.63
29.46
29.38
27.97
27.22
26.66
21.40
15.30
14.51
11.98
f1 (ppm)

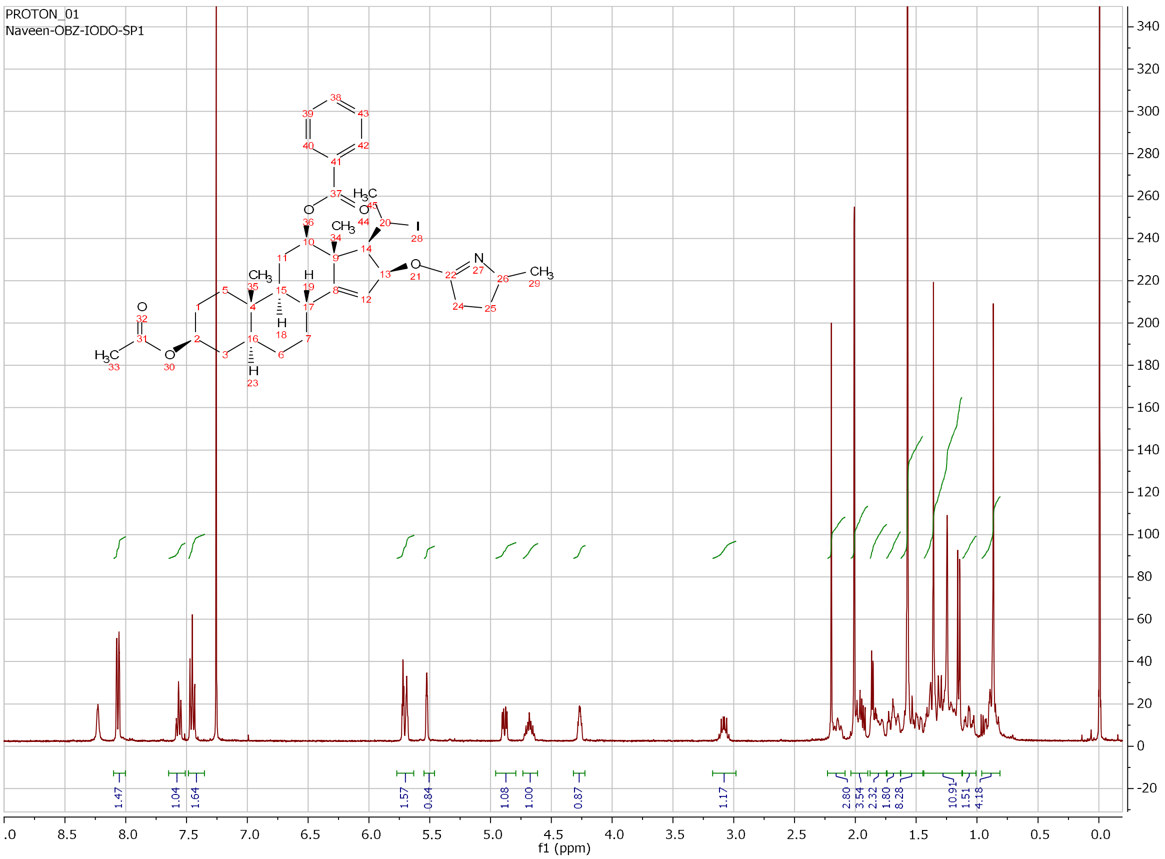

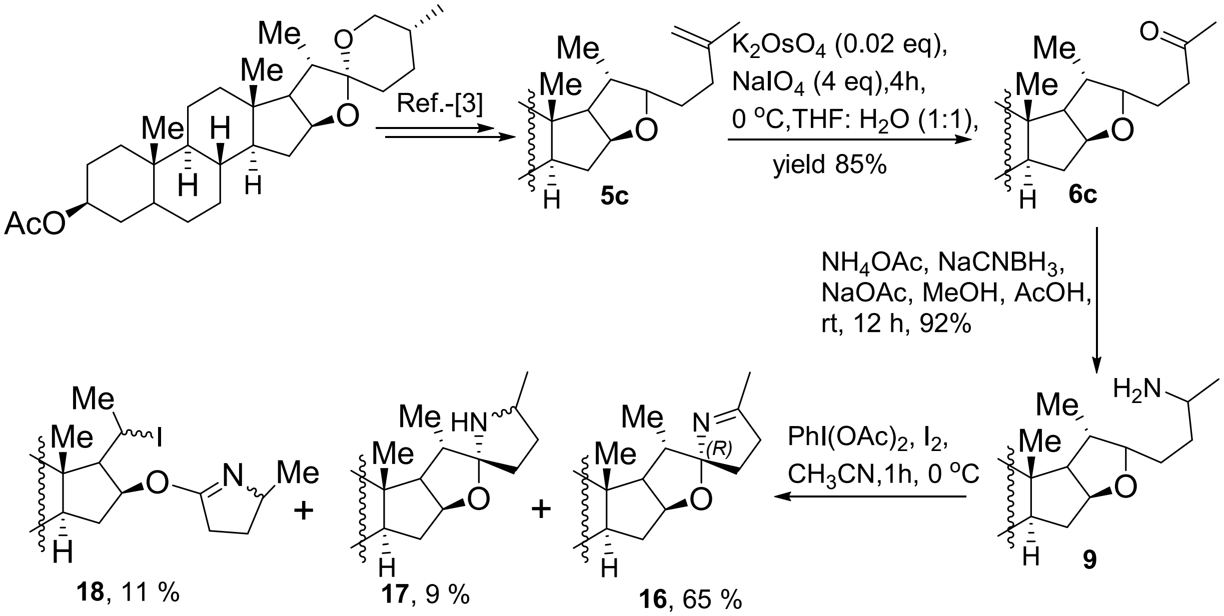

Substrates 3: Scheme S3 Complete synthesis for spiroiminal **16** formation from diosgenin acetate**.**

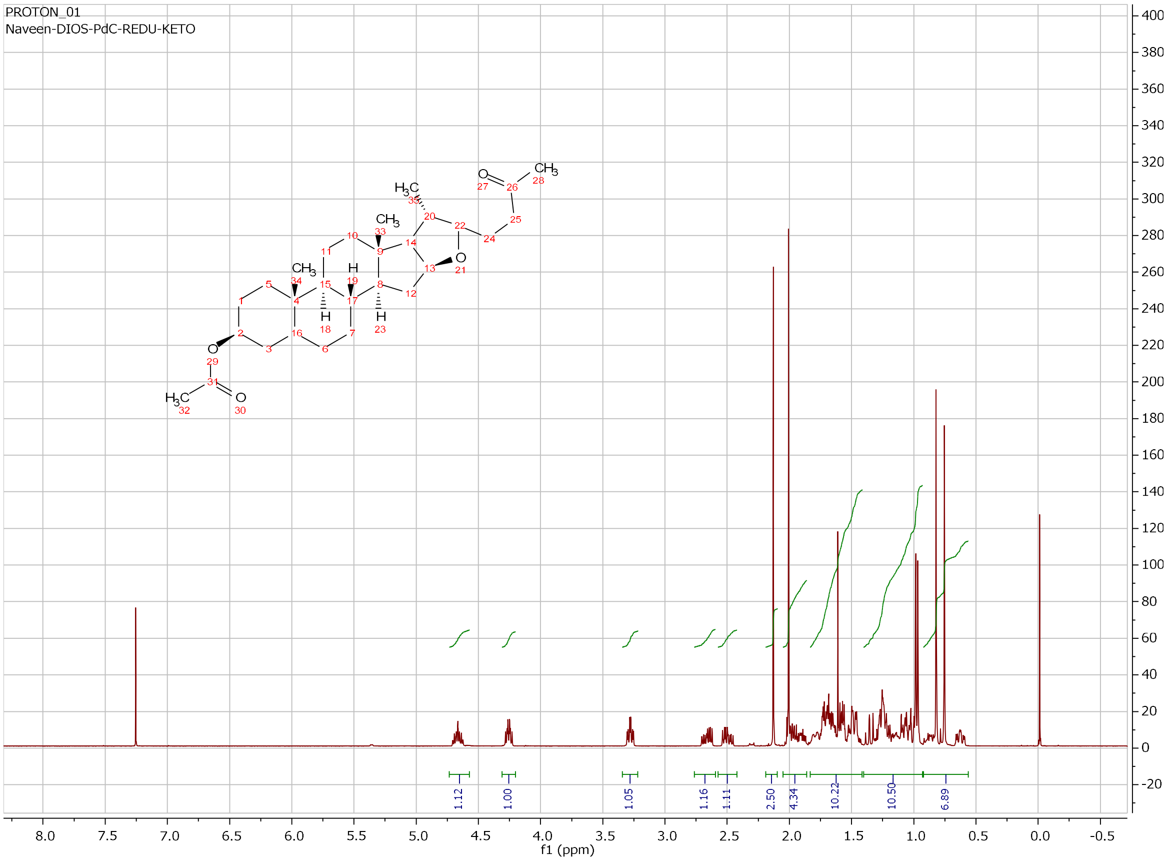
PROTON_01
Naveen-DIOS-PdC-REDU-KETO
1.12
1.00
1.05
1.16
1.11
2.50
4.34
10.22
10.50
6.89
f1 (ppm)

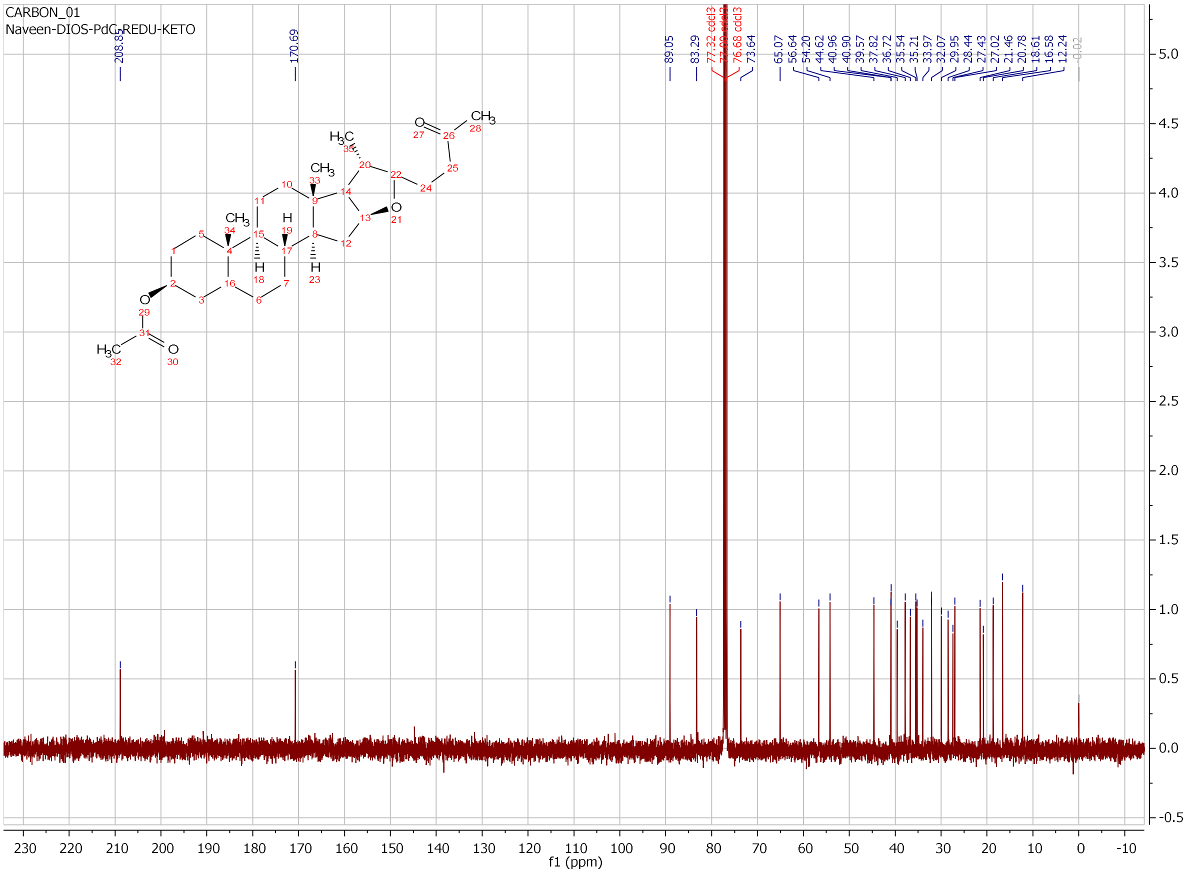
CARBON_01
Naveen-DIOS-PdC-REDU-KETO
208.83
170.69
89.05
83.29
77.32 cdcl3
76.68 cdcl3
73.64
65.07
56.64
54.20
44.62
40.96
40.90
39.57
37.82
36.72
35.54
35.21
33.97
32.07
29.95
28.44
27.43
27.02
21.46
20.78
18.61
16.58
12.24
f1 (ppm)

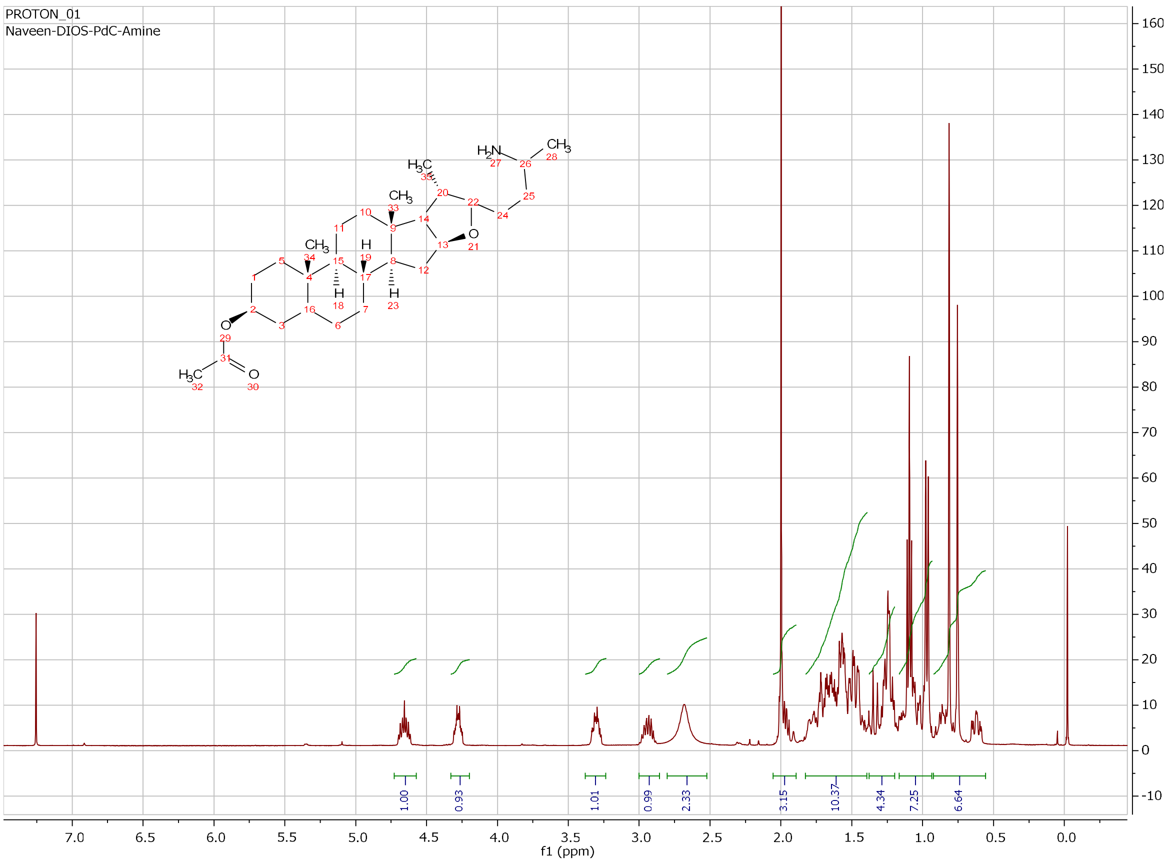
PROTON_01
Naveen-DIOS-PdC-Amine
1.00
0.93
1.01
0.99
2.33
3.15
10.37
4.34
7.25
6.64
f1 (ppm)

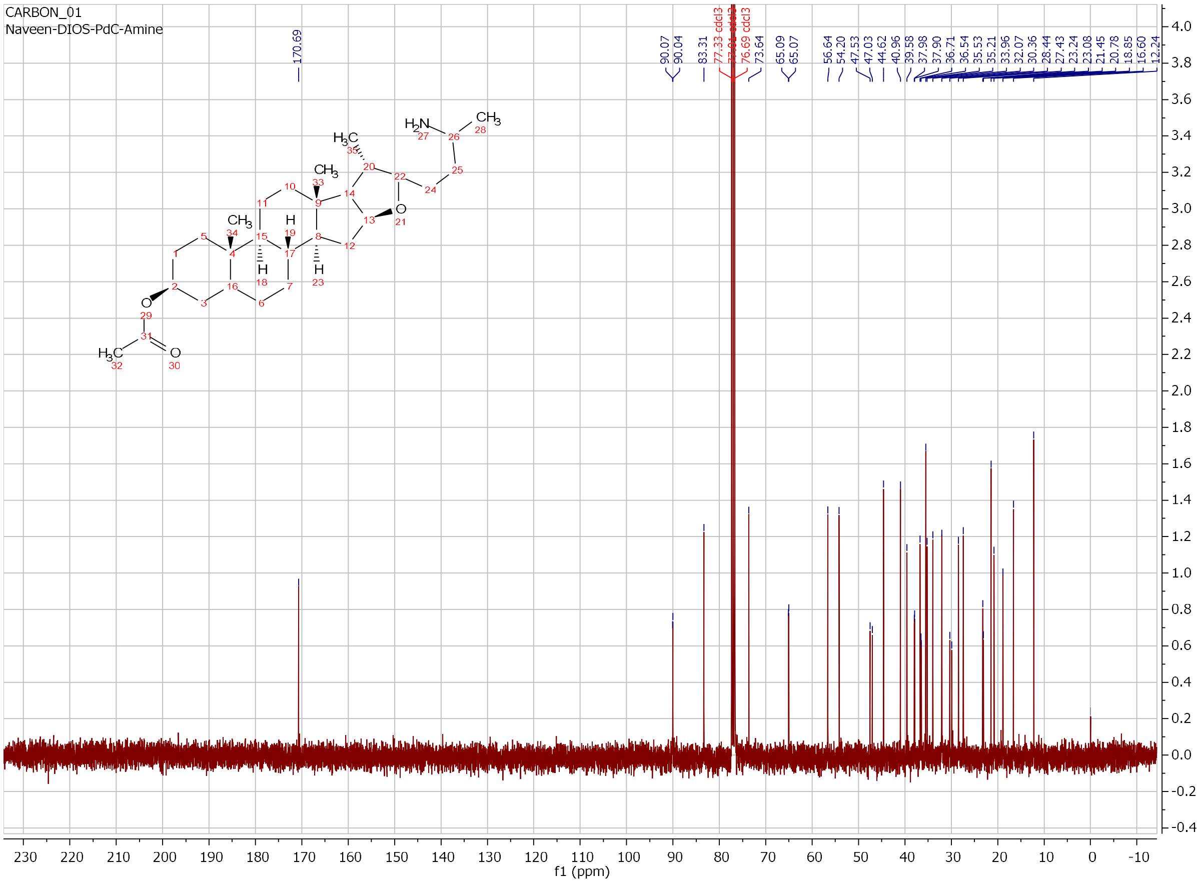
CARBON_01
Naveen-DIOS-PdC-Amine
170.69
90.07
90.04
83.31
77.33 cdcl3
76.69 cdcl3
73.64
65.09
65.07
56.64
54.20
47.53
47.03
44.62
40.96
39.58
37.98
37.90
36.71
36.54
35.53
35.21
33.96
32.07
30.36
28.44
27.43
23.24
23.08
21.45
20.78
18.85
16.60
12.24
f1 (ppm)

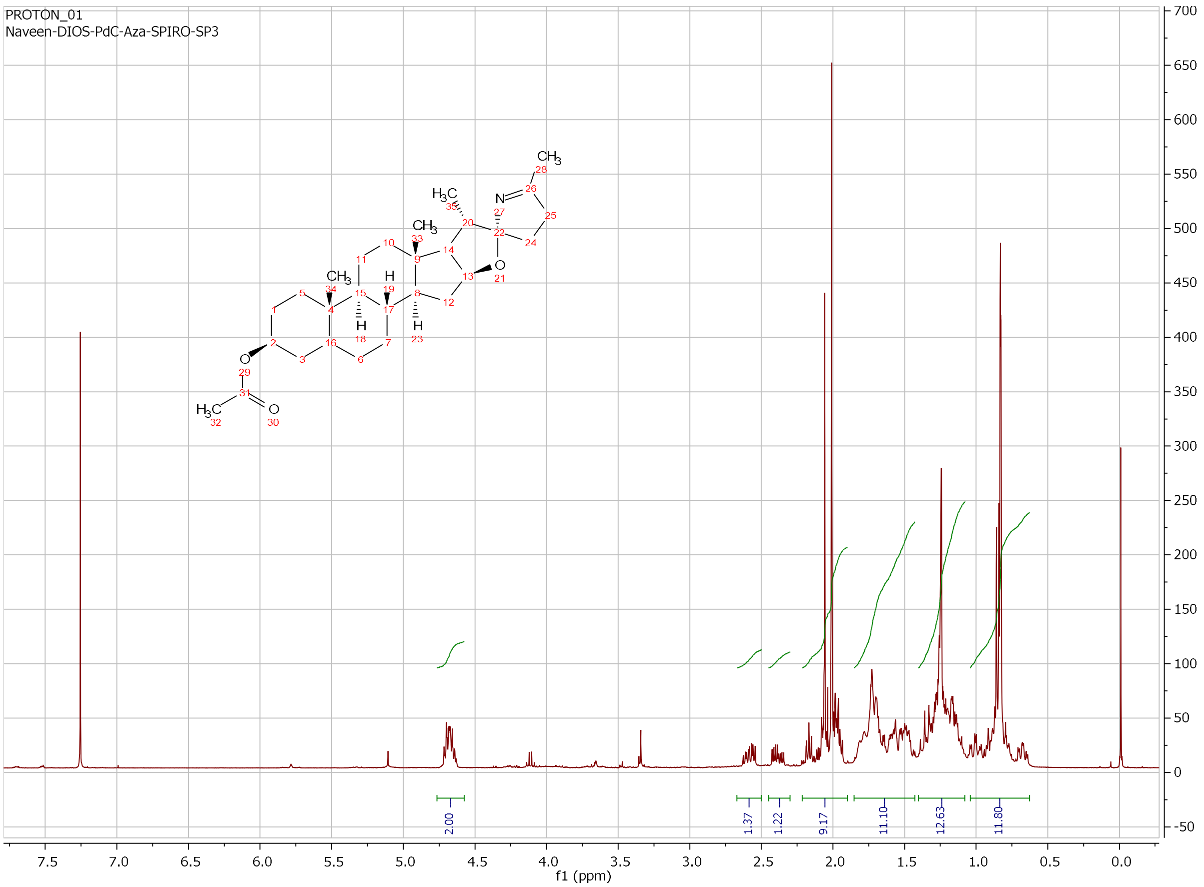
PROTON_01
Naveen-DIOS-PdC-Aza-SPIRO-SP3
f1 (ppm)
2.00
1.37
1.22
9.17
11.10
12.63
11.80

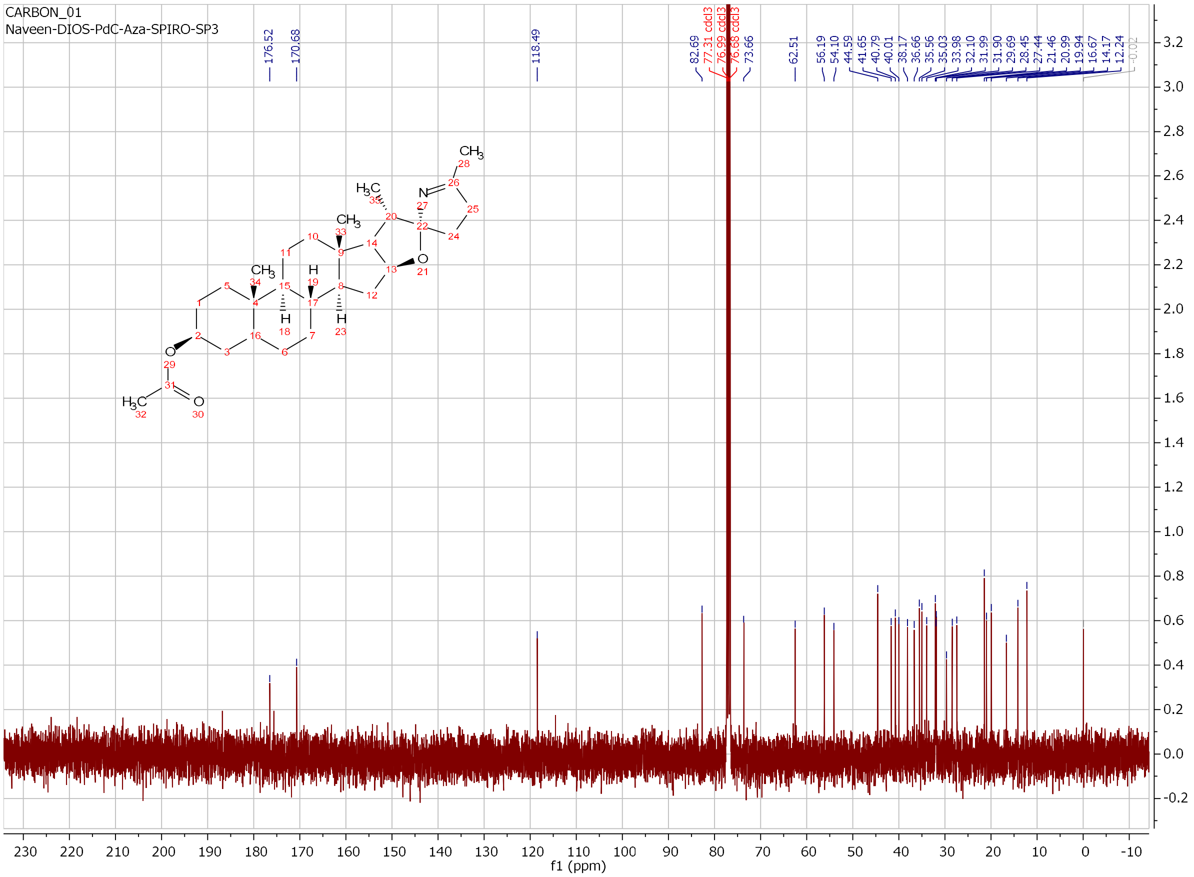
CARBON_01
Naveen-DIOS-PdC-Aza-SPIRO-SP3
176.52
170.68
118.49
82.69
77.31 cdcl3
76.99 cdcl3
76.68 cdcl3
73.66
62.51
56.19
54.10
44.59
41.65
40.79
40.01
38.17
36.66
35.56
35.03
33.98
32.10
31.99
31.90
29.69
28.45
27.44
21.46
20.99
19.94
16.67
14.17
12.24
-0.02
f1 (ppm)

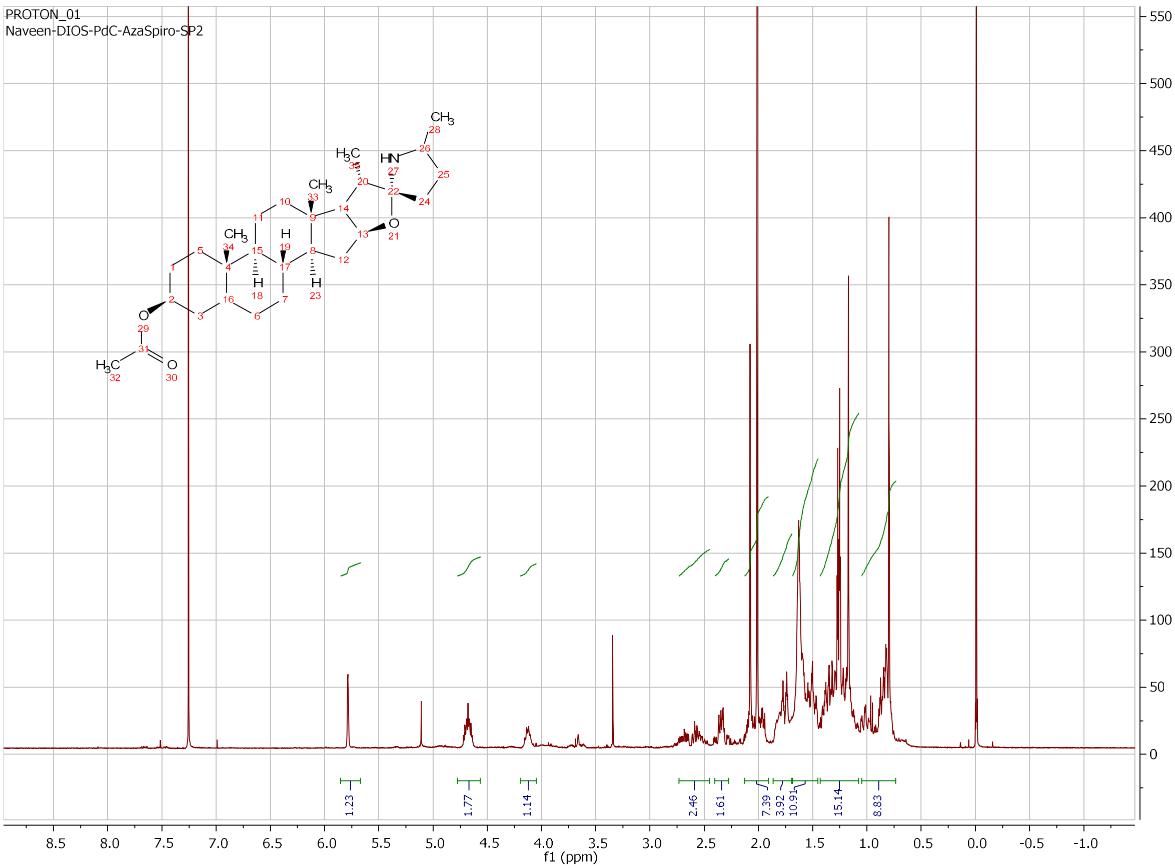
PROTON_01
Naveen-DIOS-PdC-AzaSpiro-SP2
1.23
1.77
1.14
2.46
1.61
7.39
3.92
10.91
15.14
8.83
f1 (ppm)

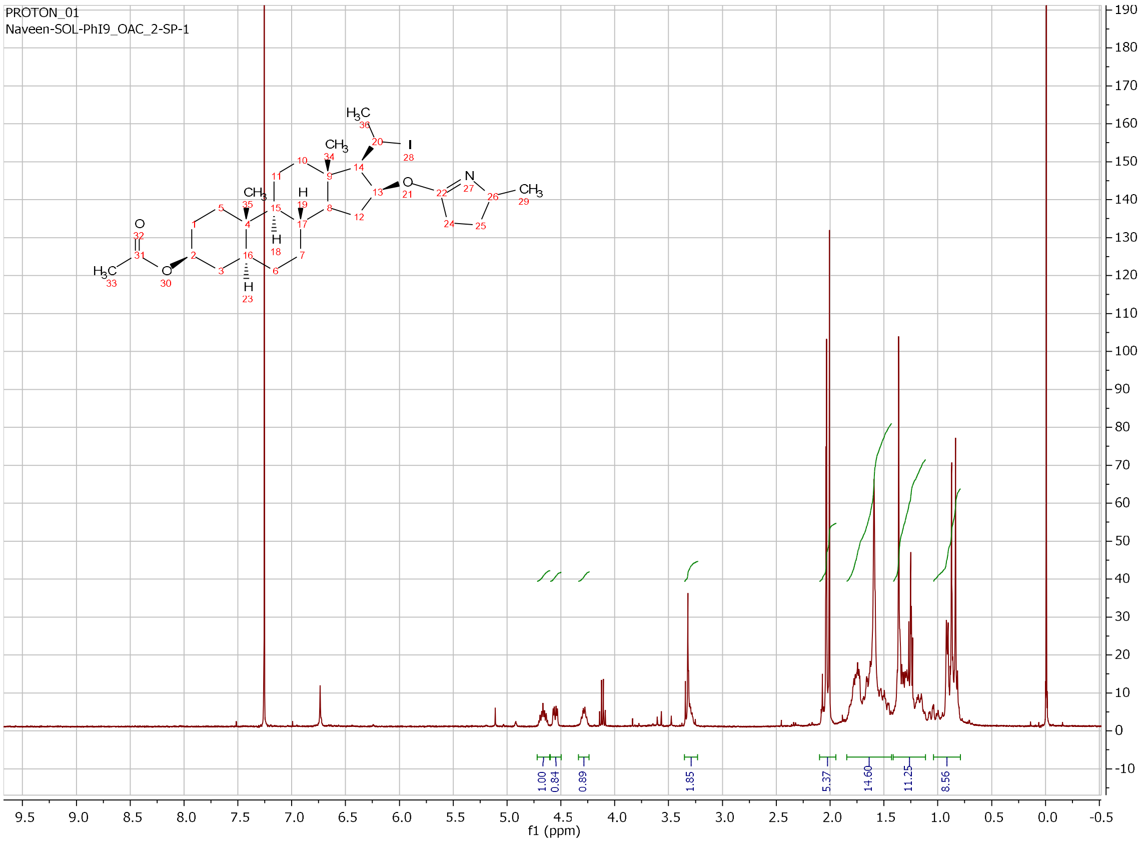
PROTON_01
Naveen-SOL-PhI9_OAC_2-SP-1
1.00
0.84
0.89
1.85
5.37
14.60
11.25
8.56
f1 (ppm)

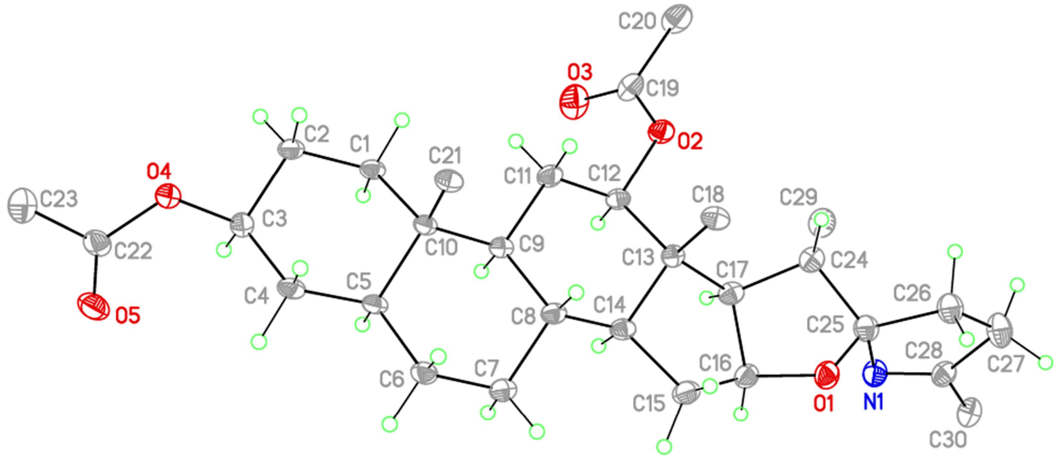

Figure S1. View of **10** showing the atom labeling scheme. Displacement ellipsoids are scaled to the 50% probability level. The methyl hydrogen atoms have been removed for clarity.

Data:

Table S1. Crystal data and structure refinement for **10**.

Empirical formula	C30 H45 N O5	
Formula weight	499.67	
Temperature	100(2) K	
Wavelength	0.71073 Å	
Crystal system	orthorhombic	
Space group	P 21 21 21	
Unit cell dimensions	a = 9.5678(9) Å	α= 90°.
	b = 11.7786(11) Å	β= 90°.
	c = 24.425(3) Å	γ = 90°.
Volume	2752.5(5) $Å^3$	
Z	4	
Density (calculated)	1.206 Mg/m^3	
Absorption coefficient	0.081 mm^{-1}	
F(000)	1088	
Crystal size	0.290 x 0.270 x 0.150 mm^3	
Theta range for data collection	2.286 to 27.568°.	
Index ranges	-12<=h<=12, -12<=k<=15, -31<=l<=31	
Reflections collected	33464	
Independent reflections	6360 [R(int) = 0.0940]	

Completeness to theta = 25.242°	99.9 %
Absorption correction	Semi-empirical from equivalents
Max. and min. transmission	1.00 and 0.735
Refinement method	Full-matrix least-squares on F^2
Data / restraints / parameters	6360 / 0 / 331
Goodness-of-fit on F^2	1.020
Final R indices [I>2sigma(I)]	R1 = 0.0460, wR2 = 0.1098
R indices (all data)	R1 = 0.0612, wR2 = 0.1178
Absolute structure parameter	-1.9(8)
Extinction coefficient	n/a
Largest diff. peak and hole	0.213 and -0.230 e.Å^{-3}

Table S2. Atomic coordinates (x 10^4) and equivalent isotropic displacement parameters ($Å^2$x 10^3) for **10**. U(eq) is defined as one third of the trace of the orthogonalized U^{ij} tensor.

	x	y	z	U(eq)
C1	5887(2)	5054(2)	6676(1)	16(1)
C2	5556(3)	5012(2)	7285(1)	17(1)
C3	5620(2)	6177(2)	7547(1)	16(1)
C4	4785(2)	7058(2)	7235(1)	17(1)
C5	5144(2)	7062(2)	6627(1)	15(1)
C6	4352(3)	7992(2)	6324(1)	17(1)
C7	4760(2)	8037(2)	5720(1)	18(1)
C8	4599(2)	6885(2)	5436(1)	15(1)
C9	5392(2)	5955(2)	5757(1)	15(1)
C10	4932(2)	5878(2)	6363(1)	14(1)
C11	5364(3)	4807(2)	5462(1)	18(1)
C12	5857(2)	4893(2)	4872(1)	15(1)
C13	4988(2)	5742(2)	4548(1)	15(1)
C14	5130(2)	6894(2)	4850(1)	16(1)
C15	4514(3)	7739(2)	4436(1)	20(1)
C16	5111(3)	7310(2)	3894(1)	18(1)
C17	5595(2)	6062(2)	3983(1)	17(1)
C18	3455(2)	5355(2)	4504(1)	18(1)
C19	6667(3)	2992(2)	4740(1)	21(1)
C20	6309(3)	1880(2)	4480(1)	30(1)
C21	3405(2)	5461(2)	6402(1)	17(1)
C22	5442(3)	6755(2)	8487(1)	20(1)
C23	4730(3)	6503(2)	9018(1)	29(1)
C24	5095(2)	5443(2)	3464(1)	18(1)
C25	4573(3)	6418(2)	3094(1)	20(1)
C26	3426(3)	6099(2)	2681(1)	24(1)
C27	4123(3)	6201(3)	2124(1)	28(1)
C28	5470(3)	6800(2)	2260(1)	21(1)
C29	6183(3)	4669(2)	3204(1)	24(1)
C30	6415(3)	7223(2)	1821(1)	25(1)
N1	5733(2)	6911(2)	2769(1)	21(1)
O1	4072(2)	7249(1)	3469(1)	20(1)

O2	5720(2)	3788(1)	4610(1)	19(1)
O3	7666(2)	3167(2)	5030(1)	32(1)
O4	5041(2)	6028(1)	8096(1)	19(1)
O5	6267(2)	7520(1)	8412(1)	26(1)

Table S3. Bond lengths [Å] and angles [°] for **10**.

C1-C2	1.520(3)	C13-C18	1.540(3)
C1-C10	1.537(3)	C13-C17	1.545(3)
C1-H1A	0.99	C13-C14	1.551(3)
C1-H1B	0.99	C14-C15	1.537(3)
C2-C3	1.516(3)	C14-H14	1.00
C2-H2A	0.99	C15-C16	1.526(3)
C2-H2B	0.99	C15-H15A	0.99
C3-O4	1.461(3)	C15-H15B	0.99
C3-C4	1.516(3)	C16-O1	1.440(3)
C3-H3	1.00	C16-C17	1.557(3)
C4-C5	1.524(3)	C16-H16	1.00
C4-H4A	0.99	C17-C24	1.539(3)
C4-H4B	0.99	C17-H17	1.00
C5-C6	1.524(3)	C18-H18A	0.98
C5-C10	1.550(3)	C18-H18B	0.98
C5-H5	1.00	C18-H18C	0.98
C6-C7	1.528(3)	C19-O3	1.207(3)
C6-H6A	0.99	C19-O2	1.342(3)
C6-H6B	0.99	C19-C20	1.496(4)
C7-C8	1.531(3)	C20-H20A	0.98
C7-H7A	0.99	C20-H20B	0.98
C7-H7B	0.99	C20-H20C	0.98
C8-C14	1.518(3)	C21-H21A	0.98
C8-C9	1.547(3)	C21-H21B	0.98
C8-H8	1.00	C21-H21C	0.98
C9-C11	1.533(3)	C22-O5	1.212(3)
C9-C10	1.546(3)	C22-O4	1.339(3)
C9-H9	1.00	C22-C23	1.494(4)
C10-C21	1.545(3)	C23-H23A	0.98
C11-C12	1.520(3)	C23-H23B	0.98
C11-H11A	0.99	C23-H23C	0.98
C11-H11B	0.99	C24-C29	1.522(3)
C12-O2	1.456(3)	C24-C25	1.543(3)
C12-C13	1.521(3)	C24-H24	1.00
C12-H12	1.00	C25-O1	1.423(3)

C25-N1	1.483(3)	C28-N1	1.276(3)
C25-C26	1.538(3)	C28-C30	1.488(4)
C26-C27	1.520(4)	C29-H29A	0.98
C26-H26A	0.99	C29-H29B	0.98
C26-H26B	0.99	C29-H29C	0.98
C27-C28	1.507(4)	C30-H30A	0.98
C27-H27A	0.99	C30-H30B	0.98
C27-H27B	0.99	C30-H30C	0.98

C2-C1-C10	112.53(19)	C6-C5-H5	107.0
C2-C1-H1A	109.1	C4-C5-H5	107.0
C10-C1-H1A	109.1	C10-C5-H5	107.0
C2-C1-H1B	109.1	C5-C6-C7	111.56(19)
C10-C1-H1B	109.1	C5-C6-H6A	109.3
H1A-C1-H1B	107.8	C7-C6-H6A	109.3
C3-C2-C1	112.04(19)	C5-C6-H6B	109.3
C3-C2-H2A	109.2	C7-C6-H6B	109.3
C1-C2-H2A	109.2	H6A-C6-H6B	108.0
C3-C2-H2B	109.2	C6-C7-C8	112.4(2)
C1-C2-H2B	109.2	C6-C7-H7A	109.1
H2A-C2-H2B	107.9	C8-C7-H7A	109.1
O4-C3-C4	110.17(18)	C6-C7-H7B	109.1
O4-C3-C2	105.30(18)	C8-C7-H7B	109.1
C4-C3-C2	112.67(19)	H7A-C7-H7B	107.9
O4-C3-H3	109.5	C14-C8-C7	112.71(19)
C4-C3-H3	109.5	C14-C8-C9	108.65(19)
C2-C3-H3	109.5	C7-C8-C9	110.42(19)
C3-C4-C5	111.95(19)	C14-C8-H8	108.3
C3-C4-H4A	109.2	C7-C8-H8	108.3
C5-C4-H4A	109.2	C9-C8-H8	108.3
C3-C4-H4B	109.2	C11-C9-C10	113.20(18)
C5-C4-H4B	109.2	C11-C9-C8	112.15(19)
H4A-C4-H4B	107.9	C10-C9-C8	112.80(19)
C6-C5-C4	111.31(19)	C11-C9-H9	106.0
C6-C5-C10	112.31(18)	C10-C9-H9	106.0
C4-C5-C10	111.90(19)	C8-C9-H9	106.0

C1-C10-C21	109.33(18)	O1-C16-C15	112.58(19)
C1-C10-C9	110.11(18)	O1-C16-C17	104.95(18)
C21-C10-C9	110.28(19)	C15-C16-C17	107.63(19)
C1-C10-C5	106.45(18)	O1-C16-H16	110.5
C21-C10-C5	112.63(19)	C15-C16-H16	110.5
C9-C10-C5	107.95(18)	C17-C16-H16	110.5
C12-C11-C9	112.46(19)	C24-C17-C13	120.33(19)
C12-C11-H11A	109.1	C24-C17-C16	103.89(19)
C9-C11-H11A	109.1	C13-C17-C16	104.06(19)
C12-C11-H11B	109.1	C24-C17-H17	109.3
C9-C11-H11B	109.1	C13-C17-H17	109.3
H11A-C11-H11B	107.8	C16-C17-H17	109.3
O2-C12-C11	109.17(18)	C13-C18-H18A	109.5
O2-C12-C13	108.09(18)	C13-C18-H18B	109.5
C11-C12-C13	111.49(19)	H18A-C18-H18B	109.5
O2-C12-H12	109.4	C13-C18-H18C	109.5
C11-C12-H12	109.4	H18A-C18-H18C	109.5
C13-C12-H12	109.4	H18B-C18-H18C	109.5
C12-C13-C18	111.30(19)	O3-C19-O2	123.7(2)
C12-C13-C17	114.75(19)	O3-C19-C20	125.5(2)
C18-C13-C17	111.5(2)	O2-C19-C20	110.9(2)
C12-C13-C14	106.30(18)	C19-C20-H20A	109.5
C18-C13-C14	112.10(19)	C19-C20-H20B	109.5
C17-C13-C14	100.30(17)	H20A-C20-H20B	109.5
C8-C14-C15	119.84(19)	C19-C20-H20C	109.5
C8-C14-C13	114.35(19)	H20A-C20-H20C	109.5
C15-C14-C13	102.70(18)	H20B-C20-H20C	109.5
C8-C14-H14	106.3	C10-C21-H21A	109.5
C15-C14-H14	106.3	C10-C21-H21B	109.5
C13-C14-H14	106.3	H21A-C21-H21B	109.5
C16-C15-C14	102.33(19)	C10-C21-H21C	109.5
C16-C15-H15A	111.3	H21A-C21-H21C	109.5
C14-C15-H15A	111.3	H21B-C21-H21C	109.5
C16-C15-H15B	111.3	O5-C22-O4	123.8(2)
C14-C15-H15B	111.3	O5-C22-C23	125.1(2)
H15A-C15-H15B	109.2	O4-C22-C23	111.2(2)

C22-C23-H23A	109.5	C28-C27-H27A	111.3
C22-C23-H23B	109.5	C26-C27-H27A	111.3
H23A-C23-H23B	109.5	C28-C27-H27B	111.3
C22-C23-H23C	109.5	C26-C27-H27B	111.3
H23A-C23-H23C	109.5	H27A-C27-H27B	109.2
H23B-C23-H23C	109.5	N1-C28-C30	123.2(2)
C29-C24-C17	114.5(2)	N1-C28-C27	115.6(2)
C29-C24-C25	115.1(2)	C30-C28-C27	121.2(2)
C17-C24-C25	103.33(19)	C24-C29-H29A	109.5
C29-C24-H24	107.9	C24-C29-H29B	109.5
C17-C24-H24	107.9	H29A-C29-H29B	109.5
C25-C24-H24	107.9	C24-C29-H29C	109.5
O1-C25-N1	109.06(19)	H29A-C29-H29C	109.5
O1-C25-C26	110.4(2)	H29B-C29-H29C	109.5
N1-C25-C26	106.17(19)	C28-C30-H30A	109.5
O1-C25-C24	104.18(19)	C28-C30-H30B	109.5
N1-C25-C24	111.24(19)	H30A-C30-H30B	109.5
C26-C25-C24	115.7(2)	C28-C30-H30C	109.5
C27-C26-C25	104.8(2)	H30A-C30-H30C	109.5
C27-C26-H26A	110.8	H30B-C30-H30C	109.5
C25-C26-H26A	110.8	C28-N1-C25	109.5(2)
C27-C26-H26B	110.8	C25-O1-C16	105.44(17)
C25-C26-H26B	110.8	C19-O2-C12	117.36(19)
H26A-C26-H26B	108.9	C22-O4-C3	117.94(18)
C28-C27-C26	102.4(2)		

Table S4. Anisotropic displacement parameters ($Å^2 x 10^3$) for **10**. The anisotropic displacement factor exponent takes the form: $-2\pi^2[h^2 a^{*2}U^{11} + ... + 2 h k a^* b^* U^{12}]$

	U^{11}	U^{22}	U^{33}	U^{23}	U^{13}	U^{12}
C1	16(1)	10(1)	20(1)	0(1)	-2(1)	1(1)
C2	19(1)	11(1)	20(1)	2(1)	-1(1)	-1(1)
C3	18(1)	17(1)	15(1)	0(1)	0(1)	-1(1)
C4	18(1)	10(1)	22(1)	-1(1)	-1(1)	2(1)
C5	15(1)	10(1)	20(1)	0(1)	-2(1)	-2(1)
C6	20(1)	12(1)	20(1)	-2(1)	-2(1)	2(1)
C7	17(1)	14(1)	21(1)	2(1)	-1(1)	0(1)
C8	14(1)	12(1)	20(1)	2(1)	-1(1)	1(1)
C9	14(1)	12(1)	18(1)	1(1)	0(1)	0(1)
C10	14(1)	11(1)	17(1)	-1(1)	-2(1)	-1(1)
C11	21(1)	14(1)	20(1)	2(1)	-1(1)	4(1)
C12	16(1)	13(1)	18(1)	1(1)	1(1)	2(1)
C13	13(1)	15(1)	17(1)	1(1)	-1(1)	0(1)
C14	13(1)	14(1)	20(1)	2(1)	0(1)	-1(1)
C15	23(1)	16(1)	22(1)	3(1)	-1(1)	1(1)
C16	17(1)	19(1)	20(1)	4(1)	-3(1)	-2(1)
C17	13(1)	17(1)	21(1)	3(1)	2(1)	-2(1)
C18	14(1)	16(1)	23(1)	-1(1)	3(1)	-1(1)
C19	24(1)	22(1)	17(1)	3(1)	5(1)	9(1)
C20	46(2)	20(1)	24(1)	-1(1)	4(1)	13(1)
C21	15(1)	14(1)	21(1)	-1(1)	1(1)	-2(1)
C22	22(1)	15(1)	22(1)	-3(1)	-3(1)	6(1)
C23	36(2)	29(1)	20(1)	-2(1)	2(1)	-1(1)
C24	15(1)	21(1)	19(1)	2(1)	2(1)	0(1)
C25	16(1)	21(1)	23(1)	0(1)	-2(1)	-1(1)
C26	18(1)	27(1)	27(1)	0(1)	-5(1)	-1(1)
C27	21(1)	38(2)	25(1)	-4(1)	-3(1)	-1(1)
C28	20(1)	21(1)	23(1)	2(1)	-3(1)	4(1)
C29	23(1)	27(1)	21(1)	0(1)	3(1)	4(1)
C30	26(1)	31(1)	20(1)	2(1)	-1(1)	2(1)
N1	19(1)	25(1)	19(1)	3(1)	-1(1)	-3(1)

O1	18(1)	22(1)	21(1)	0(1)	-4(1)	3(1)
O2	22(1)	15(1)	21(1)	0(1)	0(1)	5(1)
O3	30(1)	35(1)	30(1)	-3(1)	-4(1)	14(1)
O4	25(1)	16(1)	16(1)	0(1)	0(1)	-3(1)
O5	30(1)	18(1)	28(1)	-5(1)	1(1)	-5(1)

Table S5. Hydrogen coordinates (x 10^4) and isotropic displacement parameters ($Å^2$x 10^3) for **10**.

	x	y	z	U(eq)
H1A	5784	4283	6520	19
H1B	6872	5291	6627	19
H2A	4609	4690	7337	20
H2B	6231	4503	7469	20
H3	6616	6428	7575	20
H4A	4975	7819	7390	20
H4B	3775	6900	7280	20
H5	6161	7246	6597	18
H6A	4555	8735	6496	21
H6B	3335	7851	6355	21
H7A	5743	8291	5688	21
H7B	4166	8603	5530	21
H8	3584	6683	5431	18
H9	6394	6197	5763	18
H11A	5970	4265	5661	22
H11B	4399	4504	5468	22
H12	6860	5132	4865	19
H14	6153	7061	4871	19
H15A	3480	7713	4436	24
H15B	4824	8524	4514	24
H16	5915	7794	3776	22
H17	6639	6036	4000	20
H18A	3423	4560	4386	26
H18B	2967	5829	4235	26
H18C	2999	5431	4861	26
H20A	6662	1868	4103	45
H20B	5292	1782	4477	45
H20C	6740	1261	4689	45
H21A	3371	4643	6330	25
H21B	2833	5861	6131	25

H21C	3041	5614	6770	25
H23A	5299	6793	9321	43
H23B	4613	5681	9057	43
H23C	3812	6871	9023	43
H24	4270	4967	3565	22
H26A	3092	5315	2744	29
H26B	2622	6626	2710	29
H27A	4301	5444	1961	33
H27B	3546	6654	1868	33
H29A	5813	4357	2862	36
H29B	6402	4047	3456	36
H29C	7034	5105	3128	36
H30A	7251	7559	1987	38
H30B	5928	7799	1603	38
H30C	6689	6590	1584	38

Table S6. Torsion angles [°] for **10**.

C10-C1-C2-C3	-55.8(3)	C9-C11-C12-C13	-57.2(3)
C1-C2-C3-O4	170.16(18)	O2-C12-C13-C18	56.1(2)
C1-C2-C3-C4	50.0(3)	C11-C12-C13-C18	-63.9(3)
O4-C3-C4-C5	-167.78(18)	O2-C12-C13-C17	-71.7(2)
C2-C3-C4-C5	-50.5(3)	C11-C12-C13-C17	168.28(19)
C3-C4-C5-C6	-177.07(18)	O2-C12-C13-C14	178.39(18)
C3-C4-C5-C10	56.3(2)	C11-C12-C13-C14	58.4(2)
C4-C5-C6-C7	177.31(19)	C7-C8-C14-C15	-57.5(3)
C10-C5-C6-C7	-56.3(3)	C9-C8-C14-C15	179.74(19)
C5-C6-C7-C8	53.8(3)	C7-C8-C14-C13	179.92(19)
C6-C7-C8-C14	-174.62(19)	C9-C8-C14-C13	57.2(2)
C6-C7-C8-C9	-52.9(3)	C12-C13-C14-C8	-60.8(2)
C14-C8-C9-C11	-51.0(2)	C18-C13-C14-C8	61.0(3)
C7-C8-C9-C11	-175.12(19)	C17-C13-C14-C8	179.46(18)
C14-C8-C9-C10	179.73(18)	C12-C13-C14-C15	167.78(18)
C7-C8-C9-C10	55.6(2)	C18-C13-C14-C15	-70.4(2)
C2-C1-C10-C21	-63.1(2)	C17-C13-C14-C15	48.0(2)
C2-C1-C10-C9	175.62(19)	C8-C14-C15-C16	-169.4(2)
C2-C1-C10-C5	58.8(2)	C13-C14-C15-C16	-41.3(2)
C11-C9-C10-C1	58.8(2)	C14-C15-C16-O1	133.90(19)
C8-C9-C10-C1	-172.54(18)	C14-C15-C16-C17	18.7(2)
C11-C9-C10-C21	-62.0(2)	C12-C13-C17-C24	95.5(2)
C8-C9-C10-C21	66.7(2)	C18-C13-C17-C24	-32.2(3)
C11-C9-C10-C5	174.59(19)	C14-C13-C17-C24	-151.0(2)
C8-C9-C10-C5	-56.7(2)	C12-C13-C17-C16	-148.83(19)
C6-C5-C10-C1	174.97(18)	C18-C13-C17-C16	83.5(2)
C4-C5-C10-C1	-59.0(2)	C14-C13-C17-C16	-35.4(2)
C6-C5-C10-C21	-65.2(3)	O1-C16-C17-C24	17.4(2)
C4-C5-C10-C21	60.8(2)	C15-C16-C17-C24	137.5(2)
C6-C5-C10-C9	56.8(2)	O1-C16-C17-C13	-109.4(2)
C4-C5-C10-C9	-177.19(18)	C15-C16-C17-C13	10.7(2)
C10-C9-C11-C12	-178.44(19)	C13-C17-C24-C29	-110.4(2)
C8-C9-C11-C12	52.5(3)	C16-C17-C24-C29	133.9(2)
C9-C11-C12-O2	-176.56(18)	C13-C17-C24-C25	123.7(2)

C16-C17-C24-C25	7.9(2)	C26-C25-N1-C28	-6.9(3)
C29-C24-C25-O1	-156.68(19)	C24-C25-N1-C28	119.8(2)
C17-C24-C25-O1	-31.2(2)	N1-C25-O1-C16	-74.9(2)
C29-C24-C25-N1	-39.3(3)	C26-C25-O1-C16	168.76(19)
C17-C24-C25-N1	86.2(2)	C24-C25-O1-C16	43.9(2)
C29-C24-C25-C26	81.9(3)	C15-C16-O1-C25	-155.25(19)
C17-C24-C25-C26	-152.6(2)	C17-C16-O1-C25	-38.5(2)
O1-C25-C26-C27	129.8(2)	O3-C19-O2-C12	-5.2(4)
N1-C25-C26-C27	11.7(3)	C20-C19-O2-C12	174.8(2)
C24-C25-C26-C27	-112.2(2)	C11-C12-O2-C19	-75.3(2)
C25-C26-C27-C28	-11.7(3)	C13-C12-O2-C19	163.28(19)
C26-C27-C28-N1	8.7(3)	O5-C22-O4-C3	-0.8(3)
C26-C27-C28-C30	-171.6(2)	C23-C22-O4-C3	178.55(19)
C30-C28-N1-C25	179.0(2)	C4-C3-O4-C22	-83.8(2)
C27-C28-N1-C25	-1.2(3)	C2-C3-O4-C22	154.45(19)
O1-C25-N1-C28	-125.8(2)		
